# De novo biosynthesis of rubusoside and rebaudiosides in engineered yeasts

**DOI:** 10.1038/s41467-022-30826-2

**Published:** 2022-06-01

**Authors:** Yameng Xu, Xinglong Wang, Chenyang Zhang, Xuan Zhou, Xianhao Xu, Luyao Han, Xueqin Lv, Yanfeng Liu, Song Liu, Jianghua Li, Guocheng Du, Jian Chen, Rodrigo Ledesma-Amaro, Long Liu

**Affiliations:** 1 Key Laboratory of Carbohydrate Chemistry and Biotechnology, Ministry of Education, Jiangnan University, 214122 Wuxi, China; 2 Science Center for Future Foods, Ministry of Education, Jiangnan University, 214122 Wuxi, China; 3 Department of Bioengineering and Centre for Synthetic Biology, Imperial College London, London, SW7 2AZ UK

**Keywords:** Networks and systems biology, Synthetic biology, Metabolic engineering

## Abstract

High-sugar diet causes health problems, many of which can be addressed with the use of sugar substitutes. Rubusoside and rebaudiosides are interesting molecules, considered the next generation of sugar substitutes due to their low-calorie, superior sweetness and organoleptic properties. However, their low abundance in nature makes the traditional plant extraction process neither economical nor environmental-friendly. Here we engineer baker’s yeast *Saccharomyces cerevisiae* as a chassis for the de novo production of rubusoside and rebaudiosides. In this process, we identify multiple issues that limit the production, including rate-liming steps, product stress on cellular fitness and unbalanced metabolic networks. We carry out a systematic engineering strategy to solve these issues, which produces rubusoside and rebaudiosides at titers of 1368.6 mg/L and 132.7 mg/L, respectively. The rubusoside chassis strain here constructed paves the way towards a sustainable, large-scale fermentation-based manufacturing of diverse rebaudiosides.

## Abstract (web summary)

Rubusoside and rebaudiosides are considered the next generation of sugar substitutes. In this article, the authors report the engineering of *Saccharomyces cerevisiae*, remodelling the complex metabolic networks by a modular engineering approach, obtaining rubusoside and rebaudiosides at titers of around 1.4 g/L and 100 mg/L, respectively.

## Introduction

Considering the prevalence of non-communicable diseases (NCDs), World Health Organization published a global action plan for the prevention and control of NCDs 2013–2020^1^. In the plan, the limitation of excessive calorie intake and the reduction in the content of free and added sugars in food and beverages have been proposed. It is known that a high-caloric/sugar diet increases the risks of NCDs, including diabetes, hypertension, and kidney disease. The sweet taste is the basic taste that animals like the most^2^, but traditional sweeteners are carbohydrates with high-calorie content. Low-calorie sweeteners can be used as sugar substitutes, which can help reduce sugar consumption. In the past, because of safety concerns, artificial sweeteners like saccharine and aspartame, were questioned by consumers and never fully replaced sucrose. More recently, safe and natural sweeteners, with high sweetness and low calories such as steviol glycosides (SGs) have been proposed and commercialized^3^.

As a member of the SGs, rubusoside is about 114-fold sweeter than sucrose^4^. In addition to as a sweetener, Zhang et al. reported that rubusoside was a natural solubilizer of many anti-cancer drugs (such as botulinic acid^5^). The derivatives of rubusoside, rebaudioside A (Reb A), rebaudioside D (Reb D), and rebaudioside M (Reb M) have even higher sweetness intensities (200–350 times more than sucrose)^6^. Reb D and Reb M are especially interesting because of their better taste with no bitter aftertaste. Traditionally, rubusoside and rebaudiosides have been extracted from *Rubus suavissimus* (Chinese sweet leaf tea) and *Stevia rebaudiana*, respectively. However, their large-scale production is limited by the long growth cycle of these plants, their low titer, and their complex extraction process, which makes the production neither economical nor environmental-friendly^7^. Alternatively, synthetic biology-based microbial production, presents shorter process cycles, high efficiency, and simpler extraction processes^8,9^.

Recently, synthetic biology approaches to produce rubusoside and rebaudiosides have been developed. However, since the initial enzymatic production of rubusoside and rebaudiosides^3,10–13^ have been mainly restricted by high substrate cost, microbial de novo biosynthesis is now preferred. Wang et al. built a Reb A producer strain in *Escherichia coli*^14^, proving the concept feasible. However, synthesis efficiency was low, partially because some of the enzymes in the pathway were poorly expressed in prokaryotic cells, such as the cytochrome P450s. Compared with prokaryotic counterparts, the Generally Recognized as Safe (GRAS) chassis organism *Saccharomyces cerevisiae* is considered a superior host for expressing plant-derived P450s^15^. Olsson et al. reported that Reb D and Reb M can be synthesized in *S. cerevisiae*. They found that the UDP-dependent glycosyltransferases (UGTs) UGT76G1 is an enzyme with poor specificity and they engineer it to increase the accumulation of Reb D or Reb M, and reduce the synthesis of the unwanted side-products^16^. However, the rebaudiosides production was still low. According to previous reports, there are three main factors that limit the efficient production of rubusoside and rebaudiosides in yeast. The first one is the presence of rate-limiting steps in the biosynthesis pathway that must be overcome, to avoid that the precursor of diterpenes in the MVA pathway is insufficient, and that plant-derived P450s and UGTs exhibit low catalytic efficiencies^2,3,15^. The second factor is related to the need to improve the tolerance of yeast to rubusoside and rebaudiosides, as terpene accumulation often leads to cytotoxicity or growing pressure^17^. The third factor is related to the complexity of the metabolic network, for which it is important to identify and manipulate genetic targets to optimize it and maximize production^18^. Therefore, to develop more efficient rubusoside and rebaudiosides-producing chassis, it is necessary to address the challenges described above.

Here, we establish a *S. cerevisiae* chassis for an efficient de novo biosynthesis of rubusoside and rebaudiosides. We remodel the complex metabolic networks by a modular engineering approach, which enables the production of rubusoside and rebaudiosides at titers of 1368.6 and 132.7 mg/L in 15-L bioreactors, respectively. We engineer the substrate channeling system to improve the catalytic efficiency of the P450s. We mine and reinforce the active efflux system and stress-responding regulator to promote the secretion and synthesis of these sweeteners. In addition, we use in silico prediction tools based on genome-scale metabolic models to redistribute and optimize the metabolic networks. We expect that the engineered yeast strain here developed serves as a starting step towards the sustainable, large-scale production of rubusoside and its derivatives.

## Results

### Construction of de novo rubusoside biosynthetic pathway

**Fig. 1:**
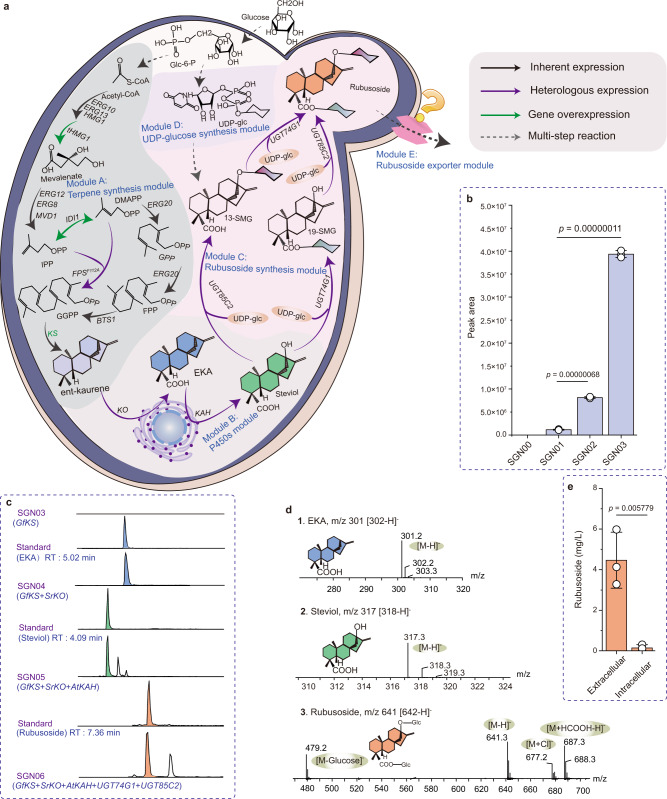
Systematic engineering of yeast metabolism for de novo biosynthesis of rubusoside. **a** Illustration of the modularized platform for producing and exporting rubusoside. Module A (terpene synthesis module) incorporates modifications designed to divert carbon flux to diterpene metabolic and brought ent-kaurene biosynthesis. Engineering yeast into the efficient platform to produce rubusoside by introducing Module B (P450s module) and Module C (rubusoside synthesis module). Module D (UDP-glucose synthesis module) provides glycoside ligands for producing rubusoside. Module E (rubusoside exporter module) is a possible exportation system of rubusoside. ERG10 acetyl-CoA C-acetyltransferase, ERG13 hydroxymethylglutaryl-CoA synthase, HMG1 hydroxymethylglutaryl-CoA reductase, tHMG1 truncated hydroxymethylglutaryl-CoA reductase, ERG12 mevalonate kinase, ERG8 phosphomevalonate kinase, IDI1 isopentenyl diphosphate delta-isomerase, *ERG20* bifunctional (2E,6E)-farnesyl diphosphate, BST1 farnesyltranstransferase, KS kaurene synthase, KO ent-kaurene oxidase, KAH kaurenoic acid 13α-hydroxylase, UGT74G1 UDP-glycosyltransferase 74G1, UGT85C2 UDP-glycosyltransferase 85C2. FPS^F112A^ mutant farnesyl pyrophosphate synthase. Glc-6-P glucose-6-phosphate, Acetyl-CoA acetyl coenzyme A, IPP isopentenyl diphosphate, GPP Geranyl diphosphate, FPP farnesyl diphosphate, GGPP geranylgeranyl pyrophosphate, DMAPP dimethylallyl diphosphate, EKA ent-kaurenoic acid, 13-SMG (5ξ,8α,9ξ,10α,13α)-13-(β-D-Glucopyranosyloxy) kaur-16-en-18-säure, 19-SMG 1-O-[(5ξ,8α,9ξ,10α,13α)-13-Hydroxy-18-oxokaur-16-én-18-yl]-β-D-glucopyranose. All the heterologous genes were controlled by *GAL* promoters. **b** Increased ent-kaurene biosynthesis by eliminating the rate-limiting steps in MVA pathway (overexpressed *tHMG1* and *IDI1*, SGN02) and avoiding competition for FPP with the monoterpene synthesis pathway (introduced *FPS*^*F112A*^, SGN03). All the heterologous genes were controlled by *GAL* promoters. **c** HPLC spectra of ent-kaurenoic acid (EKA), steviol, rubusoside, and their standards. RT retention time. **d** LC-MS analysis results of EKA, steviol, and rubusoside in negative ion mode. Source data are provided as a Source Data file. **e** The rubusoside titer difference in the intracellular and extracellular of the SGN06 strain. **b**, **e** Data are presented as mean values ± SD from three independent biological replicates (*n* = 3), the circles represent individual data points. Significance (*p*-value) was evaluated by two-sided *t*-test.

In order to build a yeast chassis with the capacity to produce high levels of rubusoside, we divided its complex metabolic pathway into engineering modules (Fig. 1a). In plant, ent-kaurene is synthesized from geranylgeranyl pyrophosphate (GGPP) by copalyl diphosphate synthases and copalyl diphosphate synthases. Interestingly, a kaurene synthase (KS) from *Gibberella fujikuroi*^19^ can directly generate ent-kaurene from GGPP. Therefore, to avoid intermediates loss caused by multi-step reactions, we firstly inserted this *KS* into the genome of *S. cerevisiae* CEN.PK2-1C, represents Module A (terpene synthesis module). In the resultant strain SGN01, ent-kaurene was detected, although at very low titers (Fig. 1b and Supplementary Fig. 1). To enhance its production, the two well-known limiting enzymes in the MVA pathway, tHMG1 (a truncated hydroxymethylglutaryl-CoA reductase^20^) and IDI1 (isopentenyl diphosphate delta-isomerase), were overexpressed^21^, resulting in strain SGN02. In addition, the precursor (farnesyl diphosphate, FPP) can be diverted towards the production of other metabolites such as ergosterol^22^, which is necessary for cell growth. GGPP is synthesized from IPP and DMAPP via GPP and FPP in *S. cerevisiae* (Fig. 1a). A mutant farnesyl pyrophosphate synthase (FPS^F112A^) was engineered to synthesize GGPP from IPP (isopentenyl diphosphate) and DMAPP (dimethylallyl diphosphate) directly^19^, which can reduce the competition for FPP. Therefore, *FPS*^*F112A*^ was introduced into SGN02 strain in order to increase the GGPP pool, creating the strain SGN03. Compared with SGN01, the area of the peak corresponding to ent-kaurene in the strains SGN02 and SGN03 increased to 7.0- and 33.9-times, respectively (Fig. 1b).

In Module B (P450s module), two P450s, KO (ent-kaurene oxidase from *S. rebaudiana*) and KAH (kaurenoic acid 13α-hydroxylase from *Arabidopsis thaliana*^23^), and one cytochrome P450s reductase (CPR1 from *S. rebaudiana*^24^) were introduced into the strain SGN03. A peak with a mass-to-charge ratio (m/z) value ([M − H]^−^ = 301.2) was detected in the strain SGN04 (KO and CPR1), and one with *m/z* value ([M − H]^−^ = 317.3) was detected in the strain SGN05 (KO, CPR1, and KAH) (Fig. 1c, Fig. 1d). Those peaks were identified as ent-kaurenoic acid (EKA) and steviol respectively when compared with the standards (Supplementary Fig. 2, Supplementary Fig. 3).

For the rubusoside synthesis module (Module C), two UDP-glycosyltransferases (UGTs) UGT74G1 and UGT85C2 from *S. rebaudiana* were integrated into the strain SGN05, generating the strain SGN06. Finally, a peak with the masses of *m/z* and mass fragmentation profiles ([M − 1 Glucose]^−^ = 479.2, [M − H]^−^ = 641.3, [M + Cl]^−^ = 677.2, and [M − H + HCOOH]^−^ = 687.3) was discovered in the strain SGN06 (Fig. 1c, Fig. 1d), which corresponded to the rubusoside standard (Supplementary Fig. 4), indicating that de novo biosynthesis pathway of rubusoside and its producer chassis strain were successfully built. The rubusoside titer was 4.5 mg/L in SGN06, and most of it was found outside of the cell (Fig. 1e). All these products of the heterologous pathway were not detected in the original strain *S. cerevisiae* CEN.PK2-1C (Fig. 1b, c).

### Identification and elimination of rate-limiting steps

**Fig. 2:**
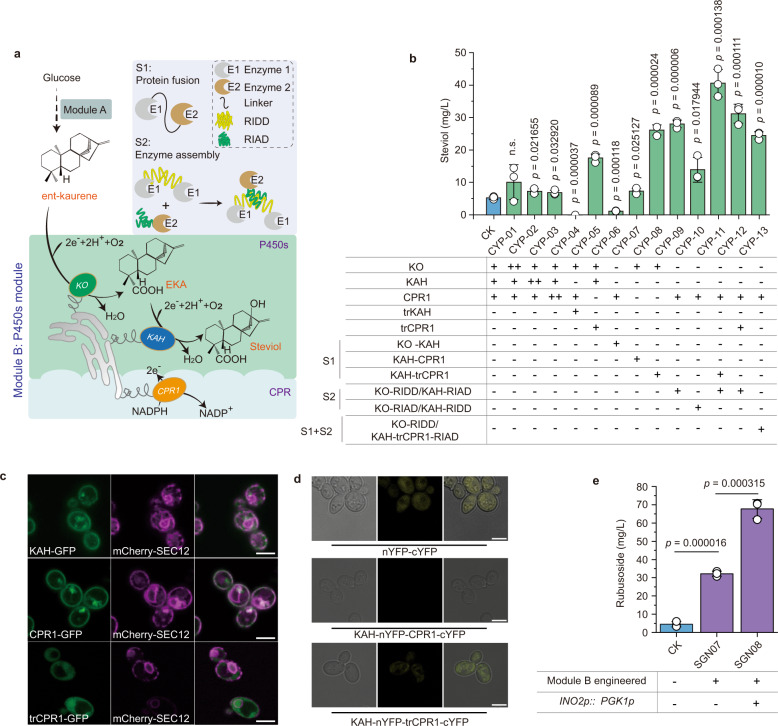
Eliminating the rate-limiting steps in the P450s module. **a** Illustration of different strategies for relieving the rate-limiting steps. S1 (Strategy1), proteins fused by a short protein linker (GGGGS_3_), S2 (Strategy2), enzymes fused with a pair of short peptide tags (RIAD and RIDD). NADPH nicotinamide adenine dinucleotide phosphate, NADP^+^ nicotinamide adenine dinucleotide phosphate. **b** Changes of steviol titer by modifying the P450s. S1, proteins fused by a linker (GGGGS_3_), S2, enzymes fused with a pair of short peptide tags (RIAD and RIDD). CK presents the SGN05 strain. **c** Visualized analysis of the subcellular localization of the P450s KAH and reductase CPR1. The fluorescence images in the first row are KAH-GFP (left, green) and mCherry-SEC12 (middle, magenta) and merge images (right). The fluorescence images in the second row are CPR1-GFP (left, green), mCherry-SEC12 (middle, magenta), and merge images (right). The fluorescence images in second row are trCPR1-GFP (left, green), mCherry-SEC12 (middle, magenta), and merge images (right). Bar = 5 μm. **d** BiFC of KAH fused with nYFP, and CPR1/trCPR1 fused with cYFP. Confocal images of the cells expressing nYFP-cYFP (top), KAH-nYFP-CPR1-cYFP (middle), and KAH-nYFP-trCPR1-cYFP (bottom). cYFP C-terminal 186–250 amino acid residues, nYFP N‐terminal 1‐185 amino acid residues. Bar = 5 μm. **e** Increased rubusoside biosynthesis by eliminating the rate-limiting steps in P450s module (Module B), and overexpressing the ER regulator INO2 by replacing it promoter (*INO2p*) with a stronger one *PGK1p*. CK (the SGN06 strain). **b**, **e** Data are presented as mean values ± SD from three independent biological replicates (*n* = 3), the circles represent individual data points. Significance (*p*-value) was evaluated by two-sided *t*-test, no significance (n.s.) presents *p* > 0.05. **b**, **c** Image analysis was carried out on the Leica LAS X software package and the ImageJ 1.53k software.

In strain SGN05, steviol titer was only 5.3 mg/L, and the titer ratio of steviol to its precursor EKA was approximately 1: 8 (Supplementary Fig. 5), indicating that synthesizing steviol from EKA is a key rate-limiting step in Module B (Fig. 2a). It is difficult to determine whether KO limits the metabolic flux from ent-kaurene to EKA, due to the lack of the commercial ent-kaurene standard. Therefore, to overcome the rate-limiting issues, all the enzymes in Module B were investigated, including the two P450s (KO and KAH) and their reductase (CPR1). First, the individual genes were integrated into SGN05. Compared with SGN05, steviol titer increased by 91.3% in CYP-01 (overexpressing *KO*), 37.1% in CYP-02 (overexpressing *KAH*), and 29.8% in CYP-03 (overexpressing *CPR1*) (Fig. 2b). Previous studies showed that plant P450s naturally anchored to the endoplasmic reticulum (ER)^25^. We found that KAH and CPR1 contain a transmembrane domain (TMD) (Supplementary Fig. 6, Supplementary Fig. 7), and anchor to the ER in yeast (Fig. 2c); and the KAH and KO are colocalized to ER (Supplementary Fig. 8). Then, to investigate whether the cytoplasmic expression of KAH and CPR1 could improve steviol synthesis, we truncated the TMD of KAH (named trKAH) and CPR1 (named trCPR1) (Fig. 2c, Supplementary Fig. 9). Surprisingly, steviol titer increased by 231.2% in CYP-05, when the TMD of CPR1 was truncated (Fig. 2b), while steviol could not be synthesized at all when the TMD of KAH was truncated (CYP-04, Fig. 2b).

To optimize the substrate trafficking, and facilitate the metabolic flux through these steps, we fused these enzymes either via a short protein linker (GGGGS_3_)^26^, or a pair of short peptide tags (RIAD and RIDD)^27^. Here, five strains were constructed; CYP-06 (KO-GGGGS_3_-KAH), CYP-07 (KAH-GGGGS_3_-CPR1), CYP-08 (KAH-GGGGS_3_-trCPR1), CYP-09 (KO-RIDD/KAH-RIAD), and CYP-10 (KO-RIAD/KAH-RIDD). Compared with SGN05, steviol titer were increased by 430.2% in CYP-09 (28.1 mg/L), 395.0% in CYP-08 (26.2 mg/L), 163.3% in CYP-10 (13.9 mg/L), and 38.9% in CYP-07 (7.4 mg/L) (Fig. 2b). The best performing CYP-09 was further engineered to generate the strains CYP-11 (KAH-GGGGS_3_-trCPR1) and CYP-12 (overexpressing trCPR1). The highest steviol titer reached 40.6 mg/L in CYP-11 (Fig. 2b). When the three enzymes were assembled together in CYP-13 (KO-RIDD/KAH-GGGGS_3_-trCPR1-RIAD), the steviol titer was only 24.5 mg/L. To visualize whether the distance between KAH and trCPR1 was reduced by the short protein linker in CYP-11, Bimolecular Fluorescence Complementation (BiFC, a method used to directly visualize protein–protein interactions^28^) assay was used. Yellow fluorescence was only observed when KAH was fused with trCPR1, but not when it was fused with CPR1 (Fig. 2d), indicating that the space between KAH and trCPR1 is shorter than that with CPR1. Besides, the subcellular localization of KAH was not affected by RIAD (Supplementary Fig. 10).

Next, to test whether the rubusoside titers change after releasing the rate-limiting step in Module B, the UGTs in Module C were integrated into the genome of CYP-11, generating the strain SGN07. In SGN07, the rubusoside titer increased to 7.2-fold from 4.5 mg/L (SGN06) to 32.2 mg/L (Fig. 2e). Furthermore, to avoid a potential imbalance between ER protein synthesis load and its folding capacity, which has been known to affect the overexpression of P450s^25^, we overexpressed the ER size regulator INO2 by replacing its endogenous promoter (*INO2p*) with a stronger one (PGK1 promoter, *PGK1p*), which generated the SGN08 strain. Rubusoside titer increased to 67.7 mg/L (SGN08, Fig. 2e).

### Improving the yeast adaptation to rubusoside by in silico prediction and experimental validation

Active efflux is a common method of adaptation to harsh environments in fungi, which is also used to export secondary metabolites and host-derived antimicrobial compounds^29^. Because most of the rubusoside was found outside the yeast cell (Fig. 1e), we speculated that an active efflux system may exist in the yeast plasma membrane (PM) to export rubusoside, which we named here Module E. We firstly screened for a rubusoside efflux pump analyzing known exporters in yeast PM, including those from the ATP-binding cassette (ABC) transporter family, the Multidrug and toxic compound extrusion (MATE) protein family, the Major Facilitator Superfamily (MFS) transporter family, and some other possibly related transporters. Their protein structures were taken from the Alpha Fold Protein Structure Database (https://alphafold.ebi.ac.uk/), and docked with rubusoside. According to our results (Supplementary Fig. 11), the affinity of rubusoside with the ABC transporters was higher than with other exporters, and rubusoside could be pulled into most of the ABC transporters channel. Thus, we speculated that ABC transporters may play vital roles in rubusoside secretion. In order to prove that, we used five common inhibitors to destroy the ABC transporters function in yeast, including reserpine, Carbonyl Cyanide m-Chlorophenylhydrazone (CCCP), PAβN, tariquidar, and dexamethasone (DMS), and we found that CCCP and reserpine can weaken the secretion of rubusoside (Supplementary Fig. 12, Supplementary Fig. 13), indicating that ABC transporters participate in the export of rubusoside.

**Fig. 3:**
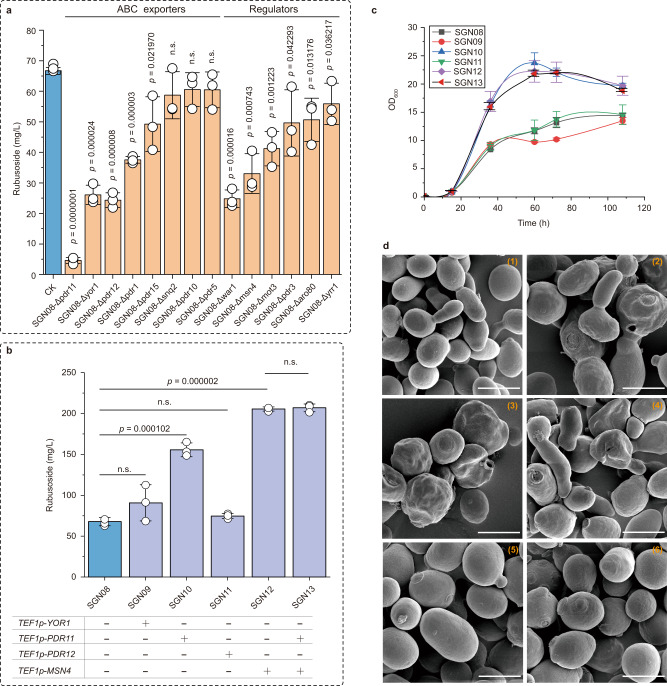
Improvement of the yeast adaptation to rubusoside. **a** Changes of rubusoside titer after deleting the ABC exporters in the PM and the pressure response regulators. Cells were cultured for 108 h; CK presents SGN08. **b** Changes of the rubusoside production in the ABC transporter and the pressure responsive factor MSN4 overexpression strains. CK presents the strain SGN08. **c** The biomass of the ABC transporter and the pressure responsive factor MSN4 overexpressed strains. **d** FESEM pictures of the yeast samples. (1). the original strain *S. cerevisiae* CEN.PK2-1C; (2). the strain SGN08; (3). the strain SGN08-Δpdr11; (4). the strain SGN08-Δmsn4. (5). the strain SGN10 (overexpressed the ABC transporter PDR11); (6). the strain of SGN12 (overexpressed stress-respond regulator *MSN4*). Strains were cultivated in shake-flask, and cells were collected after fermentation 72 h. Bar = 5 μm. **a**–**c** Data are presented as mean values ± SD from three independent biological replicates (*n* = 3). **a**, **b** The circles represent individual data points. Significance (*p*-value) was evaluated by two-sided *t*-test, n.s. presents *p* > 0.05.

Then, we found that when all known ABC transporters were individually knocked out, the rubusoside secretion titer was reduced by more than two folds in the strains SGN08-Δpdr11 (4.6 mg/L), SGN08-Δyor1 (26.1 mg/L), and SGN08-Δpdr12 (24.3 mg/L) (Fig. 3a). Additionally, the transcription levels of *PDR11* and *YOR1* increased with the rubusoside titer, which was not the case in *PDR12* (Supplementary Fig. 14, Supplementary Fig. 15). After overexpressing the three efflux pumps in the SGN08 strain using the plasmids pY16-*TEF1p*, rubusoside titer was increased by 34.0% to 90.7 mg/L in SGN09 strain (*YOR1*), 129.8% to 155.6 mg/L in SGN10 (*PDR11*), and 10.1% to 74.5 mg/L in SGN11 strain (*PDR12*) (Fig. 3b). Moreover, biomass was higher than that of strain SGN08 (Fig. 3c). In addition, the irregular cell morphology in SGN08 strain, which was caused by the inefficient export of rubusoside, returned to oval (Fig. 3d, Supplementary Fig. 16). Overall, the results indicate that PDR11 is the major efflux pump to mediate rubusoside export.

In addition, cell stress-response regulation can also trigger transport mechanisms and increase adaptation to harsh fermentation conditions in fungi^29^. Therefore, we decided to study six different stress-response factors (WAR1, MSN4, MOT3, PDR3, ARO80, and YRR1)^30–35^ in order to improve adaptation and rubusoside production. When the six genes were individually knocked out, rubusoside titer decreased by 63.6% in SGN08-Δwar1 (24.8 mg/L), 51.5% in SGN08-Δmsn4 (33.0 mg/L), 39.5% in SGN08-Δmot3 (41.2 mg/L), 27.2% in SGN08-Δpdr3 (49.6 mg/L), 25.7% in SGN08-Δaro80 (50.6 mg/L), and 18.0% in SGN08-Δyrr1 (55.9 mg/L) (Fig. 3a). This suggests that WAR1 and MSN4 may be involved in the adaptation increase to rubusoside. However, because WAR1 induces the transcription of the transporter *PDR12*, the rubusoside titer when *WAR1* was deleted (24.8 mg/L) was similar to that of SGN08-Δpdr12 (24.3 mg/L) (Fig. 3a), suggesting that the change in *PDR12* levels is the major contributor to the WAR1 deletion phenotype. Interestingly, transcription levels of *MSN4* raised with the increase in rubusoside content (Supplementary Fig. 14, Supplementary Fig. 15). When MSN4 was overexpressed using the plasmid pY16-*TEF1p* in the strain SGN08, the rubusoside titer increased by 2.0-fold to 205.5 mg/L (SGN12, Fig. 3b). As in SGN10, the cell morphology of SGN12 also reverted to normal (Fig. 3d, Supplementary Fig. 16), and biomass formation was greatly improved (Fig. 3c).

To further improve rubusoside production, the efflux-pump PDR11 and the stress-response factor MSN4 were simultaneously overexpressed in SGN08, generating the strain SGN13. However, we found that the rubusoside titer was 207.0 mg/L (SGN13, Fig. 3b), which was almost the same as in SGN12 (205.5 mg/L). Based on the RT-qPCR results, *PDR11* was upregulated by MSN4 (Supplementary Fig. 17). This suggests that transport is not limiting production and rubusoside titer can be potentially further improved by increasing the metabolic fluxes towards the rubusoside synthesis pathway.

### In silico prediction of engineering targets by genome-scale metabolic models

**Fig. 4:**
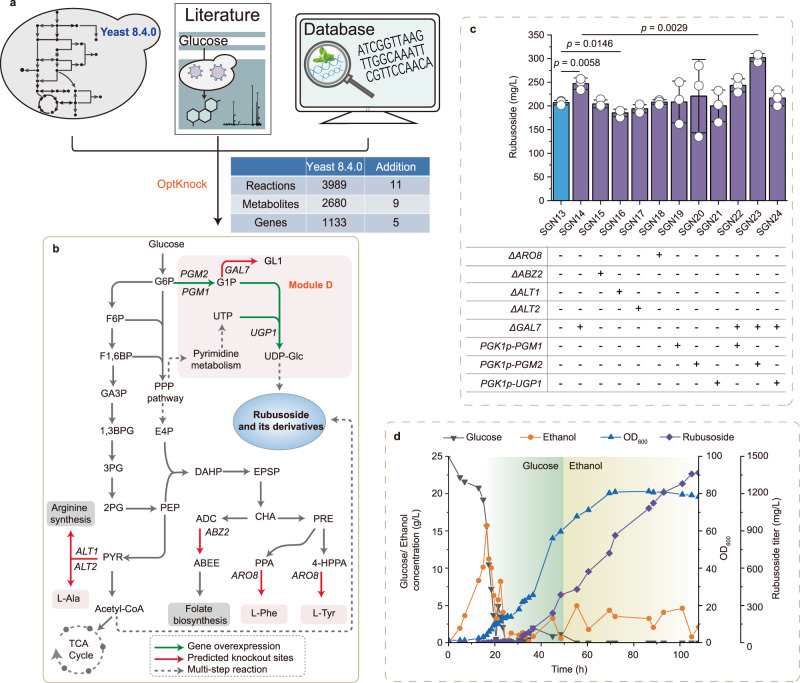
Redistribution and optimization of the metabolic networks based on in silico prediction tools. **a** Schematic illustration of the in silico prediction process. **b** Metabolic interventions predicted using OptKnock for rubusoside overproduction. GAL7 galactose-1-phosphate uridyl transferase, ABZ2 aminodeoxychorismate lyase, ALT1 alanine transaminase, ALT2 alanine transaminase, ARO8 aromatic aminotransferase I, PGM1 phosphoglucomutase, PGM2 phosphoglucomutase, UGP1 UTP (uridine triphosphate) glucose-1-phosphate uridylyltransferase. G6P glucose-6-phosphate, G1P glucose-1-phosphate, F6P fructose-6-phosphate, F1,6P fructose-1,6-bisphosphate, PPP pathway, GA3P glyceraldehyde-3-phosphate, 1,3BPG 1,3-Bisphospho-D-glycerate, 3PG 3-Phospho-D-glycerate, 2PG 2-Phospho-D-glycerate, PEP phosphoenolpyruvate, PYR pyruvate, E4P erythrose 4-phosphate, DAHP 3-deoxy-arabino-heptulonate 7-phosphate, EPSP 5-O-(1-carboxyvinyl)-3-phosphoshikimate, CHA Chorismite, PRE prephenate, ADC 4-Amino-4-deoxychorismate, ABEE 4-Aminobenzoate, PPA phenylpyruvate, L-Phe L-Phenylalanine, 4-HPPA 4-Hydroxyphenylpyruvate, L-Tyr L-tyrosine, GL1 alpha-D-Galactose-1-phosphat, UTP Uridine triphosphate, UDP-Glc UDP-glucose. **c** The changes of rubusoside titer via deleting the target genes predicted by OptKnock (SGN14–SGN18), and engineering the UDP-glucose synthesis module (SGN19–SGN24). Cells were cultured for 108 h. Data are presented as mean values ± SD from three independent biological replicates (*n* = 3), the circles represent individual data points. Significance (*p*-value) was evaluated by two-sided *t*-test. **d** Fed-batch fermentation of strain SGN23 to produce rubusoside.

To maximize the metabolic flux towards rubusoside synthesis, we combined the genome-scale metabolic model (GSMM) with the in silico prediction tool OptKnock, and determined engineering gene targets. Based on the GSMM yeast 8.4.0^36^, we first expanded the model by adding the reactions in Module A–Module C. Then, it was used for in silico simulations by constraint-based flux balance analysis (FBA)^37,38^ and knockout targets were identified by OptKnock^39^. As a result, five knockout targets were predicted (Fig. 4a, Fig. 4b); *GAL7*, *ABZ2*, *ALT1*, *ALT2*, and *ARO8*. These five genes were individually deleted in SGN13, and the resulting strains were named SGN14 (Δ*GAL7*), SGN15 (Δ*ABZ2*), SGN16 (Δ*ALT1*), SGN17 (Δ*ALT2*), and SGN18 (Δ*ARO8*). Figure 4c shows that the rubusoside titer increased by 19.4% (247.2 mg/L, SGN14) when *GAL7* was knocked out. As shown in Fig. 4b, blocking *GAL7* may favor the conversion of glucose-1-phosphate (Glc-1P) into UDP-glucose synthesis, suggesting that UDP-glucose concentration may be a rate-limiting factor for rubusoside production in SGN13. In the other knockouts, rubusoside titer was not markedly improved (Fig. 4c).

According to the literature, it is essential to supply sufficient UDP-glucose for a high biosynthesis of glycosides^15,40^. Thus, to further boost the UDP-glucose pool, we overexpressed three genes *PGM1* (phosphoglucomutase), *PGM2* (phosphoglucomutase), and *UGP1* (uridine triphosphate glucose-1-phosphate uridylyltransferase) in the SGN13 strain, using the plasmid pY13-*PGK1p*. The three generated strains were named SGN19 (overexpressing *PGM1*), SGN20 (overexpressing *PGM2*), and SGN21 (overexpressing *UGP1*). Rubusoside titer was, however, not further improved (Fig. 4c). Therefore, we decided to overexpress *PGM1*, *PGM2*, and *UGP1* in the *GAL7* deleted strain (SGN14), generating SGN22 (overexpressing *PGM1*), SGN23 (overexpressing *PGM2*), and SGN24 (overexpressing *UGP1*) strain. Surprisingly, we observed that when *PGM2* was overexpressed in SGN14 (SGN23), the rubusoside titer improved to 302.1 mg/L (Fig. 4c). In *PGM1* and *UGP1* overexpressing strains, the rubusoside titer increased to 246.8 mg/L in SGN22 and 216.9 mg/Lin SGN24. Therefore, our results further prove that the shortage of UDP-glucose is a rate-limiting step for glycoside biosynthesis. Finally, we evaluated rubusoside production of the best performing strain (SGN23) in fed-batch fermentations in a 15-L bioreactor, and the rubusoside titer reached 1368.6 mg/L (Fig. 4d), which is the highest titer achieved so far.

### Biosynthesis of rebaudiosides using the rubusoside producing chassis

**Fig. 5:**
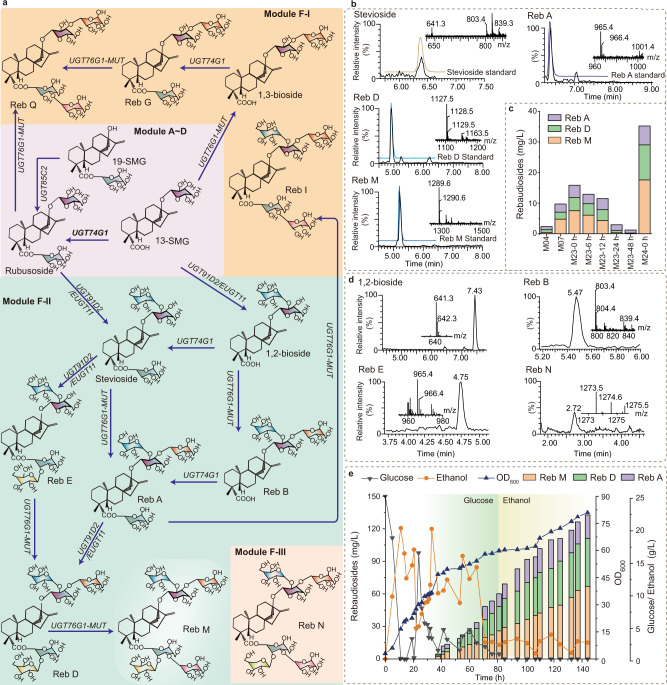
The de novo biosynthesis platform of rebaudiosides. **a** Schematic illustration of rebaudiosides biosynthesis based on the rubusoside producing strain. Module F1: the metabolic pathways were reported to exist in *S. rebaudiana*, but not be detected; Module F2: the metabolic pathways were reported to exist in *S. rebaudiana*, and the metabolites were hunted in the M23. UGT74G1 UDP-glycosyltransferase 74G1, UGT85C2 UDP-glycosyltransferase 85C2, EUGT11 UDP-glycosyltransferase 91C1, UGT76G1-MUT UDP-glycosyltransferase 76G1. 13-SMG (5ξ,8α,9ξ,10α,13α)-13-(β-D-Glucopyranosyloxy) kaur-16-en-18-säure, 19-SMG 1-O-[(5ξ,8α,9ξ,10α,13α)-13-Hydroxy-18-oxokaur-16-én-18-yl]-β-D-glucopyranose, 1,2-bioside (5β,8α,9β,10α,13α)-13-{ [2-O-(β-D-Glucopyranosyl)-β-D-glucopyran-osyl]oxy} kaur-16-en-18-oic acid, Reb A rebaudioside A, Reb B rebaudioside B, Reb C rebaudioside C, Reb D rebaudioside D, Reb E rebaudioside E, Reb G rebaudioside G, Reb I rebaudioside I, Reb M rebaudioside M, Reb N rebaudioside N, Reb Q rebaudioside Q. **b** LC-MS analysis results of stevioside, Reb A, Reb D, and Reb M in negative ion mode, analyzed by comparison with the standard product. **c** The titer changes of rebaudiosides by modifying the EUGT11 (M07), fine-turning the *UGT76G1*-*MUT* expression time (M23-0 h, M23-6 h, M23-12 h, M23-24 h, and M23-48 h) by inducing it expression an 0, 6, 12, 24, and 48 h after fermentation, and strengthening the stress-respond regulator MSN4 (M24). **d** 1,2-bioside, Reb B, Reb E, and Reb N in negative ion mode, identified by Progenesis QI v2.4 software. QI software data (Supplementary Data 1) are provided as a Source Data file. **e** Fed-batch fermentation of strain M24 to produce rebaudiosides. The *UGT76G1*-*MUT* expressed before the genes controlled by *GAL* promoters. All the data represent the mean of *n* = 3 biologically independent samples.

To explore the potential of yeast to produce rebaudiosides, we designed the rebaudioside synthesis module (Module F, Fig. 5a) to be used in the SGN08 strain. In plants, the complex metabolic networks are connected by two UGTs, UGT91D2 and UGT76G1^16^. It has been reported that substrate specificity of UGT91D2 and UGT76G1 is poor, and it is hard to convert rubusoside into rebaudiosides^41^. Previous works have reported higher catalytic activities of the mutants UGT91D2-e0e (V286A and L211M) and UGT91D2-e0w (V253I and T464A)^14^, and higher product specificity of mutant UGT76G1-MUT (T284S and I203V)^16^.

Here, we first combined the expression of the *UGT76G1-MUT* with *UGT91D2*, *UGT91D2*-*e0e*, *UGT91D2*-*e0w*, and *EUGT11* (UDP-glycosyltransferase 91C1, an isoenzyme of UGT91D2 from *Oryza sativa*^42^) in the strain SGN08. The four resultant strains were named M01 (UGT761-MUT and UGT91D2), M02 (UGT761-MUT and UGT91D2-e0e), M03 (UGT761-MUT and UGT91D2-e0w), and M04 (UGT761-MUT and EUGT11). Then, we found that the three most important rebaudiosides were detected in M04, Reb A ([M − H]^−^ = 965.4), Reb D ([M − H]^−^ = 1127.5), and Reb M ([M − H]^−^ = 1289.6) (Fig. 5b), which were verified by their corresponding standards (Supplementary Fig. 18, Supplementary Fig. 21). Although rebaudiosides were synthesized in the strain M04, all the rebaudiosides titers were lower than 1 mg/L, with low precursor titers too (Fig. 5c, Supplementary Fig. 22). After engineering EUGT11 based on the Rosetta Cartesian_ddG prediction results (M05- M22, Supplementary Fig. 23), the rebaudiosides titer increased from 2.4 mg/L (M04) to 9.1 mg/L (M07) (Fig. 5c).

To enhance the expression level and control of *UGT76G1-MUT*, we replaced the *GAL10p* with a stronger and inducible promoter *DDI2p*^43^, generating the strain M23. Here, we controlled *UGT76G1*-*MUT* by inducing its expression at different times, 0, 6, 12, 24, and 48 h. We found that rebaudiosides titers were 15.8 mg/L (M23-0 h), 12.9 mg/L (M23-6 h), 11.5 mg/L (M23-12 h), 2.9 mg/L (M23-24 h), and 1.2 mg/L (M23-48 h) (Fig. 5c). Therefore, in the following experiments, the expression of *UGT76G1*-*MUT* was induced from the beginning of the fermentation. In addition, to detect whether other types of SGs are synthesized in our rebaudiosides-producing chassis, Progenesis QI v2.4 software was used to analyze the products in the strain M23. According to the identified results, in addition to stevioside, Reb A, Reb D, and Reb M (Fig. 5b), the M23 strain was also producing, 1,2-bioside, Reb B, and Reb E (Fig. 5d). Surprisingly, Reb N, a minor SGs, whose metabolic pathway has not been yet reported, was also identified (Fig. 5d, Supplementary Data 1). Then, to further improve rebaudiosides production, the stress-responsive regulator *MSN4* was overexpressed in the M23 strain. The rebaudiosides titers in the shake-flask increased to 35.2 mg/L (M24 strain) (Fig. 5c), in which the titers of Reb A, Reb D, and Reb M were 6.2, 11.4, and 17.6 mg/L, respectively. Finally, the rebaudiosides titer was raised to 132.7 mg/L in the M24 strain using a 15-L bioreactor and a fed-batch fermentation (Fig. 5e), in which the titers of Reb A, Reb D, and Reb M were 21.5, 44.2, and 67.0 mg/L, respectively.

## Discussion

Rubusoside and rebaudiosides are important sugar substitutes with human health-related benefits^44^, which are widely used in the food, pharmaceutical, and beverage industries^6,45^. Their production using microbial cell factories would enable a faster, cheaper, and more sustainable process. Here, we proved that the *S cerevisiae* CEN.PK2-1C (without the ability to produce SGs) can be engineered to produce rubusoside and rebaudiosides from available carbon sources, such as glucose and ethanol. We demonstrated that using a modular engineering approach it is possible to produce rubusoside at a gram-per-liter level, and rebaudiosides at more than 100 mg/L, with the content of Reb D and Reb M much higher than that of Reb A.

*S. cerevisiae* has generally been considered a superior host for expressing plant-derived P450s; however, in cases with low catalytic activity or expression level, their efficiency still needs to be improved^15^. Although the catalytic activity of KAH has been improved by its co-expression with CPR1^24^, the steviol titer was still limited by the transformation of EKA into steviol (Supplementary Fig. 5). Here, we present that a substrate channeling system is an excellent strategy to enhance P450s efficiency. By this approach, the steviol titer in the strain CYP-11 reached 40.6 mg/L, with a ratio of steviol to EKA increased from 1:8 (SGN05) to 1:1.2 (Supplementary Fig. 24). In addition, the difference in steviol titer between CYP-11 and the original strain was significant (*P* < 0.001, Fig. 2b). Besides, we found that besides KAH, KO is also a rate-liming enzyme in the P450s module, because after adding KO copies steviol titer was enhanced in CYP-01, and the ent-kaurene accumulation in CYP-11 strain was decreased by 55.7% than SGN05 strain (Supplementary Fig. 25). At the same time, we demonstrated that TMD is essential for the catalytic activity of KAH, but not for CPR1. After truncating the TMD, the highest expression level of *trCPR1* was 3.0-fold higher than *CPR1* (Supplementary Fig. 26). These strategies for engineering P450s may be applied to improve the production of other natural products in microbes. In addition, they can also be used to solve common problems in the construction of other natural product chassis cells, such as the low substrate transport efficiency caused by the spatial distance of key enzymes, and the low synthesis efficiency caused by compartmentalization.

The overaccumulation of metabolites in yeast may cause metabolic or environmental stress-related problems^26,46^. To survive in complex environments, some self-protection mechanisms have evolved, such as the secretion of secondary metabolites by efflux pumps^47^. Here, because the secondary metabolites rubusoside was secreted out of the cell by an unknown active efflux mechanism (Fig. 1e), we mined the efflux pump and simulated the rubusoside exportation process using in silico prediction and molecular docking tools, which overcomes the weaknesses of the traditional method based on transcriptome analysis, more expensive and time-consuming. Using this approach, the predominant exporter of rubusoside PDR11 was identified and overexpressed, increasing the synthesis of rubusoside at the significant difference level (*P* < 0.001, Fig. 3b). On the contrary, when deleted, the rubusoside titer in vivo and vitro was significantly reduced (Supplementary Fig. 27). Our work provides effective tools and research ideas to mine exporters, which may help to increase cell tolerance to natural products. Moreover, these strategies can also be used in clinical and food security research fields to rapidly screen efflux pumps able to make the cells more sensitive to antibiotics and drugs.

Another common metabolic engineering challenge is the identification of non-obvious gene targets to optimize the metabolic network and maximize production^48^. Traditionally, the target genes need to be blindly screened from the metabolic networks^49^, but this strategy is time-consuming. GSMMs have become important tools for studying metabolism^50^, which can provide potential metabolic targets and the best metabolic engineering strategy based on model simulations. OptKnock is an algorithm that predicts knockout genes to overproduce the target products under the premise of maximizing the cell biomass^39^. With the help of OptKnock, GAL7 was identified to be involved in a competing reaction sharing the precursor Glc-1-P of UDP-glucose synthesis pathway. This indicated that the rubusoside synthesis was limited by the pool of UDP-glucose in the strain SGN13. Insufficient UDP-glucose has been identified as a key rete-liming step in glycosides production in the synthesis of daidzein^15^. Here, we demonstrate that the combination of boosting the UDP-glucose pool and disrupting GAL7, strengthened UDP-glucose synthesis and increased product formation. These results proved the importance of the glycosyl group donor UDP-glucose on glycosides production, and provided an efficient strategy for overcoming the rate-limiting step caused by insufficient UDP-glucose in the biosynthesis of natural glycosides.

Further studies can be performed to improve UGTs through protein engineering. In particular, for Reb M producing strain M24, and while the Reb M titer was higher than the other strains, rubusoside was still accumulated (158.3 mg/L, Supplementary Fig. 28). Therefore, we plan to focus future experiments on how to improve the transformation rate from rubusoside to Reb M, and enhance the specificities and purities of rebaudiosides using synthetic biology or protein engineering strategies. In this work, the GAL system, in which the transcriptional repressor Gal80 inhibits the activity of the transcriptional activator Gal4, was used to de-couple cell growth with product synthesis. After deleting Gal80, theoretically, GAL promoters can be only activated when glucose is depleted^51^. However, the modified GAL system only controlled by glucose has certain leakage of expression (Supplementary Fig. 14), which does not strictly de-couple the production and cell growth. Therefore, if a biosensor that responds to OD or the key precursors in rubusoside and rebaudiosides synthesis pathway can be successfully designed, it may be feasible to strictly de-couple the yeast growth and rubusoside and rebaudiosides production in the future.

In conclusion, although the biosynthetic pathways of rubusoside and rebaudiosides are complex, our study demonstrates that it is feasible to produce high amounts of rubusoside and rebaudiosides. Finally, the engineered strains and strategies described here have the potential to assist the engineering of complex biosynthetic pathways to produce other natural products.

## Methods

### Chemicals and reagents

All chemicals were purchased from Sangon unless otherwise specified. The ent-kaurenoic acid (EKA), steviol, rubusoside, stevioside, rebaudioside A (Reb A), rebaudioside D (Reb D), and rebaudioside M (Reb M) standards were purchased from Sigma–Aldrich. PrimeSTAR HS DNA polymerase was purchased from Takara. DNA gel purification kit and plasmid extraction kit were purchased from Thermo Scientific. Oligonucleotides were synthesized by GENEWIZ. 5-Fluoroorotic acid (5-FOA), tyrosine, histidine, leucine, and uracil were purchased from Solarbio.

### Genetic manipulation for the construction of strains and plasmids

The heterologous gene sequences used in this study are listed in Table 1 in Supplementary Data 2. The plasmids used in this work are listed in Table 2 in Supplementary Data 2. The codon-optimized genes *KS* (NCBI Accession Number: Q9UVY5), *FPS*^*F112A*^ (NCBI Accession Number: P08836.2), *KO* (NCBI Accession Number: AAQ63464.1), *KAH* (NCBI Accession Number: NP_197872.1)*, CPR1* (NCBI Accession Number: ABB88839.2), *UGT74G1* (NCBI Accession Number: Q6VAA6.1), *UGT85C2* (NCBI Accession Number: Q6VAB0.1), *UGT91D2* (NCBI Accession Number: B3VI56.1), *UGT76G1* (NCBI Accession Number: AGL95113.1), and *EUGT11* (NCBI Accession Number: XP_015629141.1) were synthesized by GenScript. All the plasmids were constructed in *E. coli* JM109 (*recA1, endA1, thi, gyrA96, supE44, hsdR17∆ (lac-proAB)/F’ [traD36, proAB*+*, lacІq, lacZ*∆*M15]*). *S. cerevisiae* CEN.PK2-1C was employed as the host for rubusoside and rebaudiosides-producing strains construction. *S. cerevisiae* CEN.PK2-1C was collected from multiple sources over the years, and stored in our lab.

The DNA manipulation was by the in vivo genetic rapid construction method^52^. All the target fragments were obtained by polymerase chain reaction (PCR) using the correct plasmid as the templates, 40–50 bp overlaps of each adjacent fragment were used to implement homologous recombination in yeast. The integration locus of chromosomal in this study was selected from the study of Reider Apel et al.^53^. The transformants with auxotroph markers were selected by the Synthetic Dropout (SD) agar plates (6.7 g/L yeast nitrogen base without amino acid, 20 g/L glucose, 50 mg/L amino acid/ uracil 200 mg/L, and 20 g/L agar). Appropriate amino acids or uracil were added to the culture medium when required (tyrosine 50 mg/L, histidine 50 mg/L, leucine 50 mg/L, or uracil 200 mg/L). After 3 days of incubation at 30 °C, the positive colonies were selected by PCR. All the primers used in this study were synthesized by GENEWIZ (Suzhou, China), and listed in Table 3 in Supplementary Data 2. All the strains used in this study are listed in Table 4 in Supplementary Data 2.

### Yeast cultivation and identification assays for production

For fermentation, the selected positive colonies were cultivated at 30 °C, 220 rpm in 50 mL culture tubes containing 5 mL SD liquid medium. After 16–18 h incubation, cultures were transferred into 250 mL shake-flask with 25 mL YPD medium (20 g/L tryptone, 10 g/L yeast extract, and 20 g/L glucose), and cultivated at 30 °C, 220 rpm for 96 h (EKA and steviol production strains), 108 h (rubusoside production strains), or 144 h (rebaudiosides production strains).

The ent-kaurene, EKA, and steviol were extracted from the cell cultures using a mixture extractant (2-propanol: n-hexane = 1: 2) in a 4: 1 ratio (organic: liquid, v/v). The mixture was vigorously shaken with a vortex mixer for 10 min and then centrifuged at 5000 × *g* for 5 min to separate the two phases. The supernatant was transferred to sample vials for measurements. For extracting the rubusoside and its derivatives SGs in intracellular, the cell precipitate was broken by a high-pressure homogenizer (UH-06, Union), then the mixture was centrifuged at 5000 × *g* for 5 min to separate the supernatant. The fermentation supernatant was used to analyze the concentration of rubusoside and SGs extracellular.

The ent-kaurene were detected by GC-MS on GC-MS-QP2010Ultra equipped with an HP-5MS column (30 m × 0.25 mm × 0.25 μm, Shimadzu). The GC-MS operational condition was as follows: initial temperature 80 °C for 1 min, followed by a temperature ramp of 15 °C/min to 245 °C, a ramp of 5 °C/min to 300 °C; the flow rate of helium was 1.2 mL/min.

In the biosynthesis pathway from ent-kaurene to rebaudiosides, we constructed two methods with LC-MS/MS to analyze them more accurately. Waters (MALDI SYNAPT MS) equipped with BEH C18 BA column was used to detect EKA and steviol. Samples were eluted at 30 °C with the following gradient program with solvent A (acetonitrile) and solvent B (0.1% formic acid) as the mobile phase at a flow rate of 0.3 mL/ min: 70% solvent B (0–0.1 min), 40% solvent B (0.1–4 min), 0% solvent B (4–6 min), 0% solvent B (6–7 min), 70% solvent B (7–7.1 min). The BSH column (150 × 2 mm^2^, 3 μm, Waters) was used to detect the rubusoside and its derivatives. Samples were eluted at 35 °C with the following gradient program with solvent A (acetonitrile) and solvent B (0.1% formic acid) as the mobile phase at a flow rate of 0.3 mL/min: 85% solvent B (0–0.1 min), 50% solvent B (0.1–10 min), 0% solvent B (10–12 min), 85% solvent B (12–12.1 min). MS was set as electrospray ionization (ESI)-negative mode. Full MS was set as follows: 100–1500 *m/z*. According to the LC-MS/MS results, the Progenesis QI v2.4 (Waters) was used to obtain reliable and definitive Identity Documents (IDs) of the SGs types.

### Image analysis and fluorescence detection

The green fluorescent protein (GFP) was severally fused to the C-terminus of the genes *KAH*, *CPR1*, and *trCPR1* by a fusion protein linker (GGGGS_3_), and the expression cassette was inserted into pY16-*TEF1p* plasmid through using Gibson Assembly® Cloning Kit (NEB). The red fluorescence protein (mCherry) was fused with the residues 355–479 of the protein SEC12, which anchored to the ER in yeast^54^, and the expression cassette was inserted into pY14-*TEF1p* plasmid through Gibson assembly. To confirm the subcellular localization of KAH and CPR1 in yeast, the object proteins and the ER marker SEC12 (the C-terminal domain of SEC12, which includes a single TMD specifying ER localization) were individually labeled by GFP and mCherry.

Bimolecular Fluorescence Complementation (BiFC) was used to perform the spatial distance of KAH with CPR1 and trCPR1. The complete yellow fluorescent protein (YFP) was divided into two non-fluorescent fragments: nYFP (the N-terminal 1–185 amino acid residues) and cYFP (C-terminal 186–250 amino acid residues)^28^. nYFP and cYFP were fused with KAH with CPR1 or trCPR1 fragments, respectively. Then two fusion fragments containing nYFP and cYFP were fused by a linker, and the expression cassette was inserted into pY16-*TEF1p* plasmid through Gibson assembly. When their space is close enough, the yellow fluorescence will be emitted by 488 nm laser line.

The fluorescent protein GFP, mCherry, and YFP were excited by 488, 595, and 510 nm laser lines of an argon-ion laser line of a He–Ne laser, respectively. The fluorescence proteins were observed by Leica microscope (Leica, Mannheim, Germany) fitted with a ×60 oil-immersion objective. Fluorescence emissions were detected with spectral detector sets BP 500–525 for GFP, BP 600–625 for mCherry, and BP 520–555 for YFP. Image analysis was carried out on the Leica LAS X software package and the Image J 1.53k software (National Institutes of Health, Bethesda, MA).

### Field emission scanning electron microscopy (FESEM)

After fermentation 72 h in YPD, the fresh samples were centrifuged to remove the supernatant and were stored at 4 °C. First, samples were fixated with 5% glutaraldehyde then those were rinsed with 0.1 M phosphate buffer solution five times. Next, the samples were dehydrated by ethanol with a gradient concentration of 30, 50, 70, 90, and 100%. Following, after the critical point drying (CPD-300, LEICA) and ion sputtering (ACE-600, LEICA), the samples can be observed by cold field emission scanning electron microscope observation (SU8220, HITACHI)

### Flux balance analysis

The model used in this study with some modification is based on the model yeast 8.4.0 (https://github.com/SysBioChalmers/yeast-GEM/releases). The yeast 8.4.0 model was used and deletions of the *YEL021W*, *YCL018W*, *YDR007W*, and *YOR202W* genes were simulated by setting their corresponding reactions flux bounds to zero. The amino acid uptake rate of the model was set to be 0.1 mmol/g DCW h^−1^. The model was manually curated to be functional for rubusoside production. To represent the Rubusoside biosynthesis pathway, the following reactions were added to the model: ‘geranylgeranyl diphosphate → ent-kaurene + diphosphate’, ‘ent-kaurene + NADPH + O_2_ → NADP^+^ + H_2_O + ent-kaurenoic acid’, ‘ent-kaurenoic acid + NADPH + O_2_ → NADP^+^ + H_2_O + steviol’, ‘steviol + UDP-D-glucose → H^+^ + UDP + 13-SMG’, ‘steviol + UDP-D-glucose → H^+^ + UDP + 19-SMG’, ’13-SMG + H^+^ + UDP-D-glucose→ formaldehyde + UDP + Rubusoside’ and ’19-SMG + H^+^ + UDP-D-glucose→ formaldehyde + UDP + Rubusoside’. The reconstructed model was subsequently used for in silico simulations. The in silico simulations were performed by FBA, which is a constraint-based flux analysis method^37,38^. For analysis of rubusoside production, the knockout algorithms OptKnock^39^ were used to identify knockout targets. The glucose uptake rate of the model was set to be 20 mmol/g DCW h^−1^. The synthesis reaction of rubusoside, r_4638, was set as an objective function to identify the potential targets. The in silico simulations were performed using COBRA Toolbox v3.0^55^ with Gurobi Optimizer (Gurobi Optimization Inc., Houston TX) in Matlab 2019b (The Mathworks Inc., Natick, MA, USA).

### Fed-batch fermentation in 15-L bioreactor

Seed culture was carried out in 500 mL shake-flask containing 100 mL of YPD medium at 30 °C with shacking at 220 rpm for 24 h. The seed cultures were inoculated (10%, v/v) into a 15- L bioreactor (T&J-Dtype; T&J Bio-engineering Co., Ltd) with a 12 L medium. The fermentation medium contained 20 g/L yeast extract, 40 g/L tryptone, and 25 g/L glucose. The agitation speed was adjusted from 300 to 800 rpm with a constant air input flow rate of 1.5 vvm, and NH_4_OH was automatically added to maintain the pH at 5.5. Feeding was conducted using a two-stage strategy. In the first stage, the feeding solution consisting of 500 g/L glucose, 9 g/L KH_2_PO_4_, 2.5 g/L MgSO_4_, 3.5 g/L K_2_SO4, 0.28 g/L Na_2_SO_4_, 10 mL/L trace metal solution, and 12 mL/L vitamin solution^21^ was used to sustain fast cell growth. In the second stage, when the strain entered the late-logarithmic growth phase, the feeding solution was changed to 400 g/L ethanol to support rubusoside and its derivatives accumulation. In the feeding stage, the feeding speed was automatically controlled by dissolved oxygen-stat (DO-stat), in which the glucose and ethanol concentration was controlled at no more than 5 g/L. The fermentation temperature was 30 °C.

### Statistical analysis

All experiments were independently carried out at least three times, and the results were expressed as mean ± standard deviation (SD). All the statistical evaluation (*p*-value) was performed by a two-sided *t*-test in Excel (Microsoft Office 365), *p* > 0.05 is presented by no significance (n.s.).

### Reporting summary

Further information on research design is available in the Nature Research Reporting Summary linked to this article.

## Supplementary information

Supplementary Information

Description of Additional Supplementary Files

Supplementary Data 1

Supplementary Data 2

Reporting Summary

## Data Availability

The authors declare that all data supporting the findings of this study are available in the article and its supplementary files or are available from the corresponding author on request. Sequence data in this article can be found in the National Coalition Building Institute (NCBI) under the accession codes and links of DNA sequences presented in the Source Data file. All the protein structures of efflux pumps in this study have been predicted previously and are obtained from Alpha fold; all the links of efflux-pump protein structure are listed in Supplementary Fig. 11 in Source Data file. The source data for all figures reported in the article and its supplementary information is provided in the Source Data file. The metabolomics results of the M23 strain are provided in Supplementary Data 1. Heterologous gene sequences, plasmids, primers, and strains used in this work are listed in the Supplementary Table 1–4, provided in the Supplementary Data 2 file. Source data are provided with this paper.

## Source data

Source Data

## References

[1] World Health Organization. *Global action plan for the prevention and control of noncommunicable diseases 2013–2020*. (World Health Organization, 2013).

[2] Yang T (2019). Hydrophobic recognition allows the glycosyltransferase UGT76G1 to catalyze its substrate in two orientations. Nat. Commun..

[3] Zhang J (2021). Catalytic flexibility of rice glycosyltransferase OsUGT91C1 for the production of palatable steviol glycosides. Nat. Commun..

[4] Zhang Y (2020). Efficient biocatalytic preparation of rebaudioside KA: highly selective glycosylation coupled with UDPG regeneration. Sci. Rep..

[5] Zhang J, Chou G, Liu Z, Liu M (2016). Employing rubusoside to improve the solubility and permeability of antitumor compound betulonic acid. Nanomedicine.

[6] Chen L (2018). Synthesis of rebaudioside D, using glycosyltransferase UGTSL2 and in situ UDP-glucose regeneration. Food Chem..

[7] Zhou YJ (2012). Modular pathway engineering of diterpenoid synthases and the mevalonic acid pathway for miltiradiene production. J. Am. Chem. Soc..

[8] Nielsen J, Keasling JD (2016). Engineering cellular metabolism. Cell.

[9] Lee SY (2019). A comprehensive metabolic map for production of bio-based chemicals. Nat. Catal..

[10] Sun Y (2018). Diterpenoid UDP-glycosyltransferases from Chinese sweet tea and Ashitaba complete the biosynthesis of rubusoside. Mol. Plant..

[11] Shu W (2020). Enhanced heterologous production of glycosyltransferase UGT76G1 by co-expression of endogenous *prpD* and *malK* in *Escherichia coli* and its transglycosylation application in production of rebaudioside. Int. J. Mol. Sci..

[12] Mao Y, Chen Z, Ren Y, Sun Y, Wang Y (2021). Whole-cell biocatalyst for rubusoside production in *Saccharomyces cerevisiae*. J. Agric. Food Chem..

[13] Chen L (2020). Production of rebaudioside D from stevioside using a UGTSL2 Asn358Phe mutant in a multi-enzyme system. Microb. Biotechnol..

[14] Wang J, Li S, Xiong Z, Wang Y (2016). Pathway mining-based integration of critical enzyme parts for *de novo* biosynthesis of steviolglycosides sweetener in *Escherichia coli*. Cell. Res..

[15] Liu Q (2021). *De novo* biosynthesis of bioactive isoflavonoids by engineered yeast cell factories. Nat. Commun..

[16] Olsson K (2016). Microbial production of next-generation stevia sweeteners. Microb. Cell. Fact..

[17] Lv Y (2019). Coupling feedback genetic circuits with growth phenotype for dynamic population control and intelligent bioproduction. Metab. Eng..

[18] Xu X (2020). Pyruvate-responsive genetic circuits for dynamic control of central metabolism. Nat. Chem. Biol..

[19] Geiselman GM (2020). Production of ent-kaurene from lignocellulosic hydrolysate in *Rhodosporidium toruloides*. Microb. Cell. Fact..

[20] Zhao Y, Zhang Y, Nielsen J, Liu Z (2021). Production of β-carotene in *Saccharomyces cerevisiae* through altering yeast lipid metabolism. Biotechnol. Bioeng..

[21] Westfall PJ (2012). Production of amorphadiene in yeast, and its conversion to dihydroartemisinic acid, precursor to the antimalarial agent artemisinin. Proc. Natl Acad. Sci. USA.

[22] Zhao J, Bao X, Li C, Shen Y, Hou J (2016). Improving monoterpene geraniol production through geranyl diphosphate synthesis regulation in *Saccharomyces cerevisiae*. Appl. Microbiol. Biotechnol..

[23] Moon JH, Lee K, Lee JH, Lee PC (2020). Redesign and reconstruction of a steviol-biosynthetic pathway for enhanced production of steviol in *Escherichia coli*. Microb. Cell. Fact..

[24] Gold ND (2018). A combinatorial approach to study cytochrome P450 enzymes for *de novo* production of steviol glucosides in baker’s yeast. Acs. Synth. Biol..

[25] Kim J-E (2019). Tailoring the *Saccharomyces cerevisiae* endoplasmic reticulum for functional assembly of terpene synthesis pathway. Metab. Eng..

[26] Qin J (2021). Engineering yeast metabolism for the discovery and production of polyamines and polyamine analogues. Nat. Catal..

[27] Kang W (2019). Modular enzyme assembly for enhanced cascade biocatalysis and metabolic flux. Nat. Commun..

[28] Lv X (2020). Synthetic metabolic channel by functional membrane microdomains for compartmentalized flux control. Metab. Eng..

[29] Coleman JJ, Mylonakis E (2009). Efflux in fungi: la piece de resistance. PLoS Pathog..

[30] Kren A (2003). War1p, a novel transcription factor controlling weak acid stress response in yeast. Mol. Cell. Biol..

[31] Martínez-Montañés F, Rienzo A, Poveda-Huertes D, Pascual-Ahuir A, Proft M (2013). Activator and repressor functions of the Mot3 transcription factor in the osmostress response of *Saccharomyces cerevisiae*. Eukaryot. Cell..

[32] Delahodde A, Delaveau T, Jacq C (1995). Positive autoregulation of the yeast transcription factor Pdr3p, which is involved in control of drug resistance. Mol. Cell. Biol..

[33] Pereira R (2020). Elucidating aromatic acid tolerance at low pH in *Saccharomyces cerevisiae* using adaptive laboratory evolution. Proc. Natl Acad. Sci. USA.

[34] Rong-Mullins X, Ayers MC, Summers M, Gallagher JEG (2018). Transcriptional profiling of *Saccharomyces cerevisiae* reveals the impact of variation of a single transcription factor on differential gene expression in 4NQO, fermentable, and nonfermentable carbon sources. G3.

[35] Li X (2021). Metabolic network remodelling enhances yeast’s fitness on xylose using aerobic glycolysis. Nat. Catal..

[36] Lu H (2019). A consensus *S. cerevisiae* metabolic model Yeast8 and its ecosystem for comprehensively probing cellular metabolism. Nat. Commun..

[37] Toya Y, Shimizu H (2013). Flux analysis and metabolomics for systematic metabolic engineering of microorganisms. Biotechnol. Adv..

[38] Orth JD, Thiele I, Palsson BØ (2010). What is flux balance analysis?. Nat. Biotechnol..

[39] Burgard AP, Pharkya P, Maranas CD (2003). Optknock: a bilevel programming framework for identifying gene knockout strategies for microbial strain optimization. Biotechnol. Bioeng..

[40] Zhuang Y (2017). Biosynthesis of plant-derived ginsenoside Rh2 in yeast via repurposing a key promiscuous microbial enzyme. Metab. Eng..

[41] Chen L (2017). A fusion protein strategy for soluble expression of Stevia glycosyltransferase UGT76G1 in *Escherichia coli*. 3 Biotech.

[42] Wang Z (2020). Heterologous expression of EUGT11 from *Oryza sativa* in *Pichia pastoris* for highly efficient one-pot production of rebaudioside D from rebaudioside A. Int. J. Biol. Macromol..

[43] Wang Y (2019). Fine-tuning the expression of target genes using a DDI2 promoter gene switch in budding yeast. Sci. Rep..

[44] Ullah A, Munir S, Mabkhot Y, Badshah SL (2019). Bioactivity profile of the diterpene isosteviol and its derivatives. Molecules.

[45] Zhao L (2020). Enzymatic monoglucosylation of rubusoside and the structure-sweetness/taste relationship of monoglucosyl derivatives. J. Agric. Food Chem..

[46] Chen R, Yang S, Zhang L, Zhou YJ (2020). Advanced strategies for production of natural products in yeast. iScience.

[47] Cassemiro NS (2021). New derivatives of the iridoid specioside from fungal biotransformation. Appl. Microbiol. Biotechnol..

[48] Somerville V, Grigaitis P, Battjes J, Moro F, Teusink B (2022). Use and limitations of genome-scale metabolic models in food microbiology. Curr. Opin. Food Sci..

[49] Cui S (2019). Engineering a bifunctional *Phr60*-*Rap60*-*Spo0A* quorum-sensing molecular switch for dynamic fine-tuning of menaquinone-7 synthesis in *Bacillus subtilis*. Acs. Synth. Biol..

[50] Lieven C (2020). MEMOTE for standardized genome-scale metabolic model testing. Nat. Biotechnol..

[51] Ai L, Guo W, Chen W, Teng Y, Bai L (2019). The gal80 Deletion by CRISPR-Cas9 in engineered *Saccharomyces cerevisiae* produces artemisinic acid without galactose induction. Curr. Microbiol..

[52] Shao Z, Zhao H, Zhao H (2009). DNA assembler, an in vivo genetic method for rapid construction of biochemical pathways. Nucleic Acids Res..

[53] Reider Apel A (2017). A Cas9-based toolkit to program gene expression in *Saccharomyces cerevisiae*. Nucleic Acids Res..

[54] Sato M, Sato K, Nakano A (1996). Endoplasmic reticulum localization of Sec12p is achieved by two mechanisms: Rer1p-dependent retrieval that requires the transmembrane domain and Rer1p-independent retention that involves the cytoplasmic domain. J. Cell. Biol..

[55] Heirendt L (2019). Creation and analysis of biochemical constraint-based models using the COBRA Toolbox v.3.0. Nat. Protoc..

