# Supplementary material for: De novo biosynthesis of rubusoside and rebaudiosides in engineered yeasts
Source: Nat Commun. 2022 Jun 1;13:3040. doi: 10.1038/s41467-022-30826-2 (PMC9160076; doi:10.1038/s41467-022-30826-2)
Supplement: Supplementary file 1 — Supplementary Information [file 41467_2022_30826_MOESM1_ESM.pdf]

Supplementary Information

De novo biosynthesis of rubusoside and rebaudiosides in engineered yeasts

Yameng Xu[a,b], Xinglong Wang[a,b], Chenyang Zhang[a,b], Xuan Zhou[a,b], Xianhao Xu[a,b], Luyao Han[a,b], Xueqin Lv[a,b], Yanfeng Liu[a,b], Song Liu[a,b], Jianghua Li[a,b], Guocheng Du[a,b], Jian Chen[a,b], Rodrigo Ledesma-Amaro[c], Long Liu [a,b†]

[a]Key Laboratory of Carbohydrate Chemistry and Biotechnology, Ministry of Education, Jiangnan University, Wuxi, 214122, China

[b]Science Center for Future Foods, Ministry of Education, Jiangnan University, Wuxi, 214122, China.

[c]Department of Bioengineering and Centre for Synthetic Biology, Imperial College London, London SW7 2AZ, UK

†Corresponding author: Long Liu, Tel: +86-0510-85918312, Fax: +86-0510-85918309, E-mail: longliu@jiangnan.edu.cn.

Supplementary Figures:

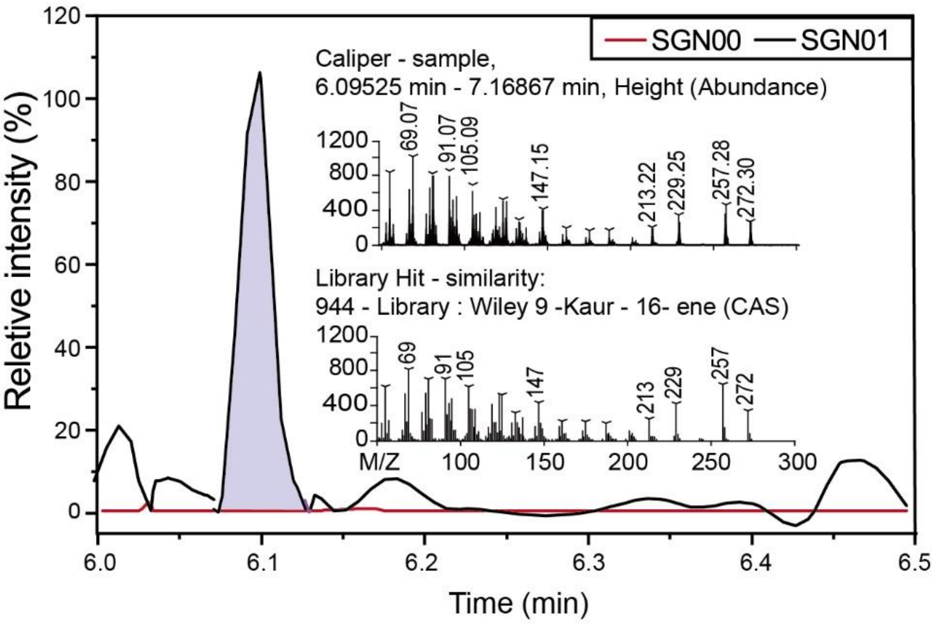

Supplementary Figure 1. The GC-MS results of ent-kaurene in the SGN01 strain. The m/z of ent-kaurene is 272. SGN00 is the control strain (original *S. cerevisiae* CEN.PK2-1C).

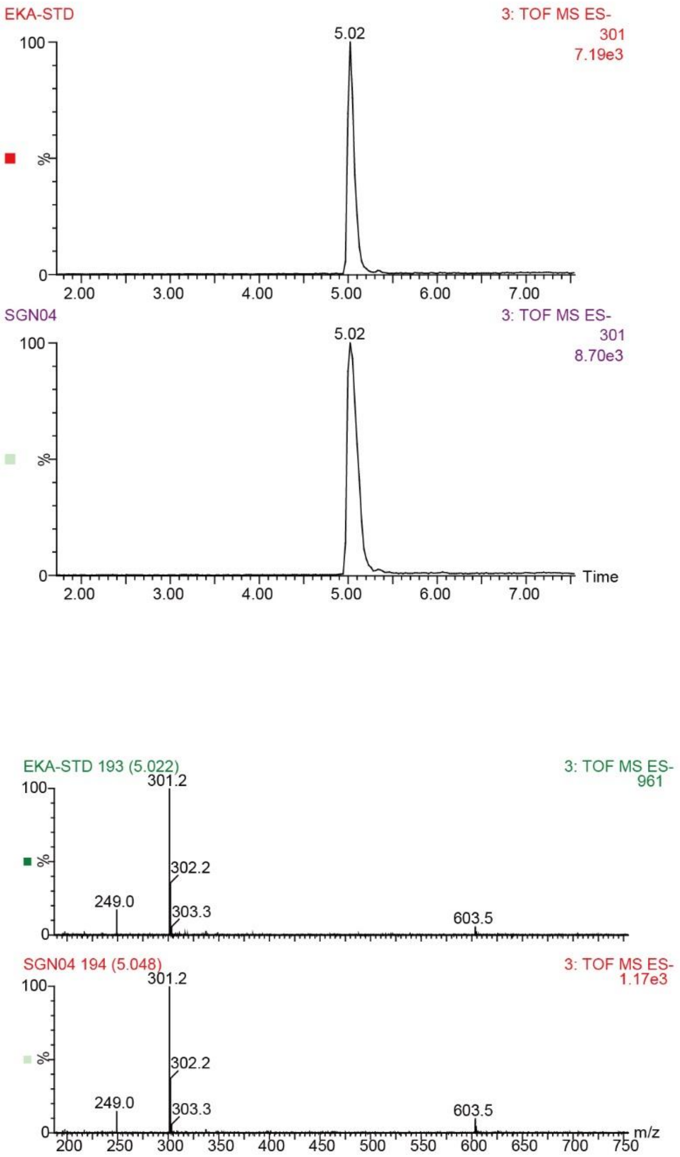

Supplementary Figure 2. The LC-MS/MS results of SGN04 strain. a. HPLC spectra of ent-kaurenoic acid (EKA) and the standard. b. MS/MS analysis results of EKA and the standard in negative ion mode. The m/z of EKA is 301.2.

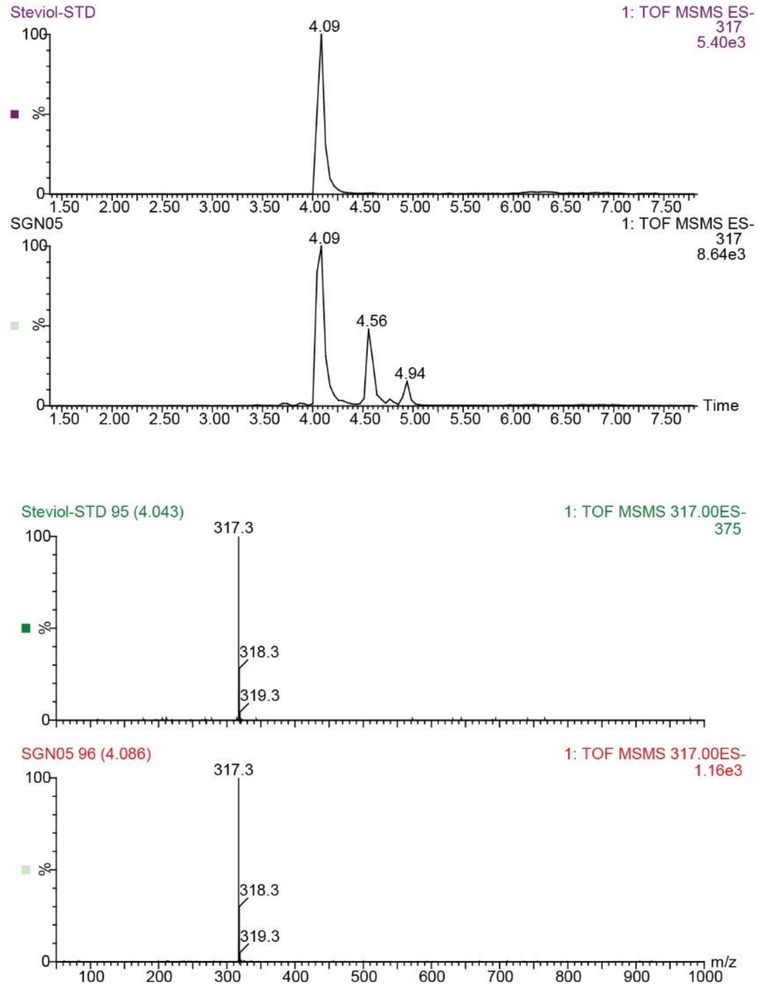

Supplementary Figure 3. The LC-MS/MS results of SGN05 strain. a. HPLC spectra of steviol and the standard. b. MS/MS analysis results of steviol and the standard in negative ion mode. The m/z of steviol is 317.3.

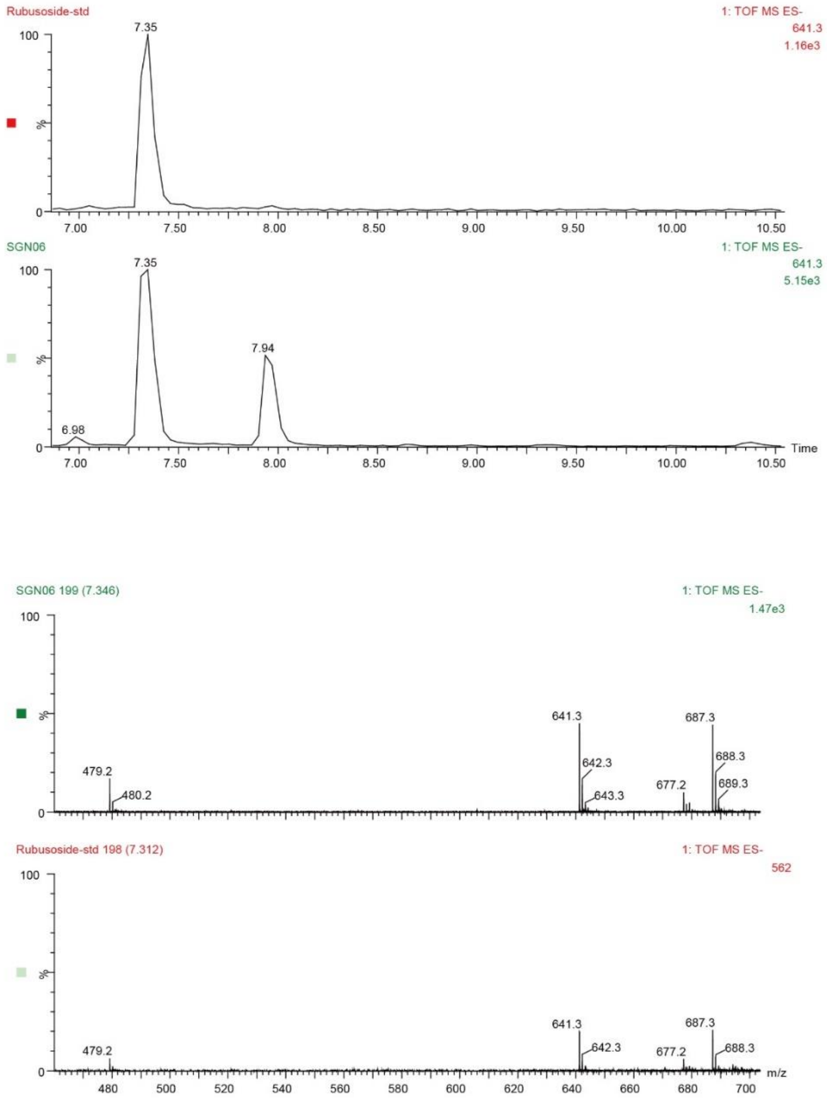

Supplementary Figure 4. The LC-MS/MS results of SGN06 strain. a. HPLC spectra of rubusoside and the standard. b. MS/MS analysis results of rubusoside and the standard in negative ion mode. The m/z of rubusoside is 641.3.

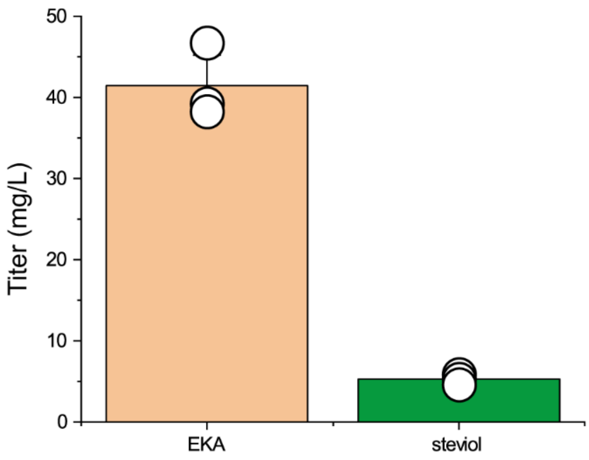

Supplementary Figure 5. The titers of EKA and steviol in the strain SGN05. Those results indicate that the P450 enzyme KAH is a rate-liming step to transform EKA into steviol. Three experiments (n = 3) were repeated independently with similar results. Data are presented as mean values ± SD.

TMHMM result

```
# WEBSEQUENCE Length: 525
# WEBSEQUENCE Number of predicted TMHs:  1
# WEBSEQUENCE Exp number of AAs in TMHs: 22.52801
# WEBSEQUENCE Exp number, first 60 AAs:  21.84959
# WEBSEQUENCE Total prob of N-in:        0.93179
# WEBSEQUENCE POSSIBLE N-term signal sequence
WEBSEQUENCE     TMHMM2.0        inside       1     6
WEBSEQUENCE     TMHMM2.0        TMhelix      7    29
WEBSEQUENCE     TMHMM2.0        outside     30   525
```

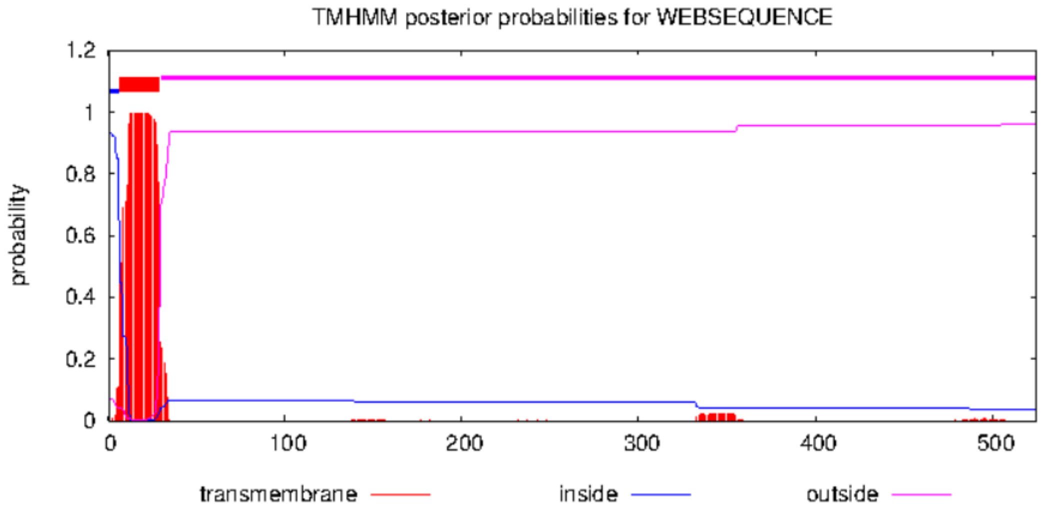

plot in postscript, script for making the plot in gnuplot, data for plot

Supplementary Figure 6. Prediction of transmembrane helices of the cytochrome P450 enzyme KAH (kaurenoic acid 13α-hydroxylase) from *Arabidopsis thaliana* predicted in the TMHMM - 2.0.

TMHMM result

```
# WEBSEQUENCE Length: 710
# WEBSEQUENCE Number of predicted TMHs:  1
# WEBSEQUENCE Exp number of AAs in TMHs: 20.57712
# WEBSEQUENCE Exp number, first 60 AAs:  11.72858
# WEBSEQUENCE Total prob of N-in:        0.97999
# WEBSEQUENCE POSSIBLE N-term signal sequence
WEBSEQUENCE     TMHMM2.0        inside       1    48
WEBSEQUENCE     TMHMM2.0        TMhelix     49    69
WEBSEQUENCE     TMHMM2.0        outside     70   710
```

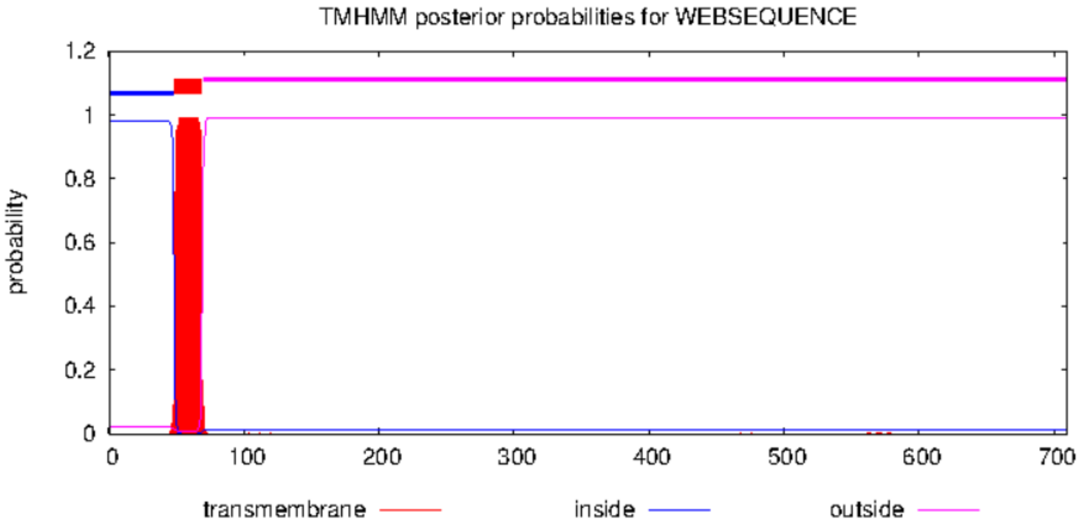

plot in postscript, script for making the plot in gnuplot, data for plot

Supplementary Figure 7. Prediction of transmembrane helices of CPR1 (cytochrome P450s reductase) from *Stevia rebaudiana* predicted in the TMHMM - 2.0.

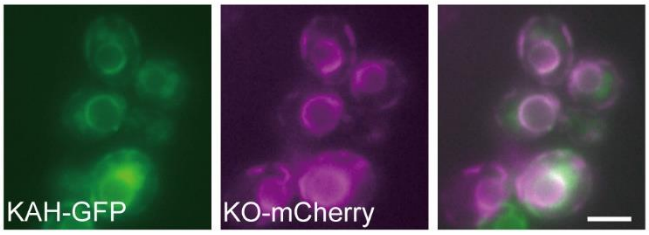

Supplementary Figure 8. Co-localization of KAH-GFP (green) and KO-mCherry (magenta).

The fluorescence images in the left is KAH‑GFP, KO-mCherry (middle), and merge images (right). Bar = 5 μm. Three experiments (n = 3) were repeated independently with similar results. Image analysis was carried out on the Leica LAS X software package and the ImageJ 1.53k software.

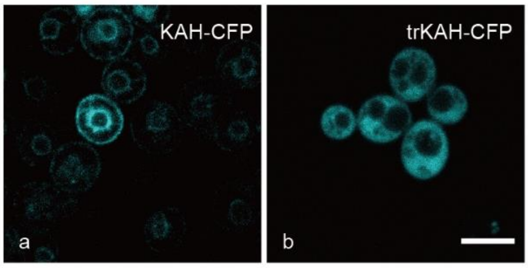

Supplementary Figure 9. Fluorescence microscopy images of KAH-CFP and trKAH-CFP.

trKAH: KAH truncated the N-terminus signal peptide (2 - 29 aa). Bar = 5 μm. Three experiments (n = 3) were repeated independently with similar results. Image analysis was carried out on the Leica LAS X software package and the ImageJ 1.53k software.

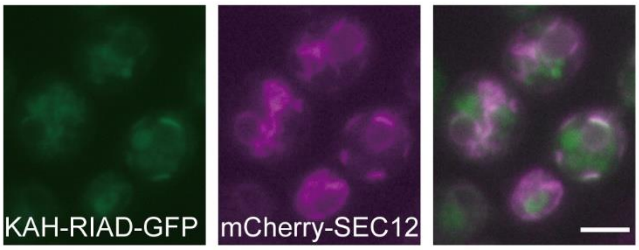

Supplementary Figure 10. Fluorescence microscopy images of KAH-RIAD-GFP (green) and SEC12-mCherry (magenta). Bar = 5 µm. Three experiments (n = 3) were repeated independently with similar results. Image analysis was carried out on the Leica TCS LAX S software package and the ImageJ 1.53k software.

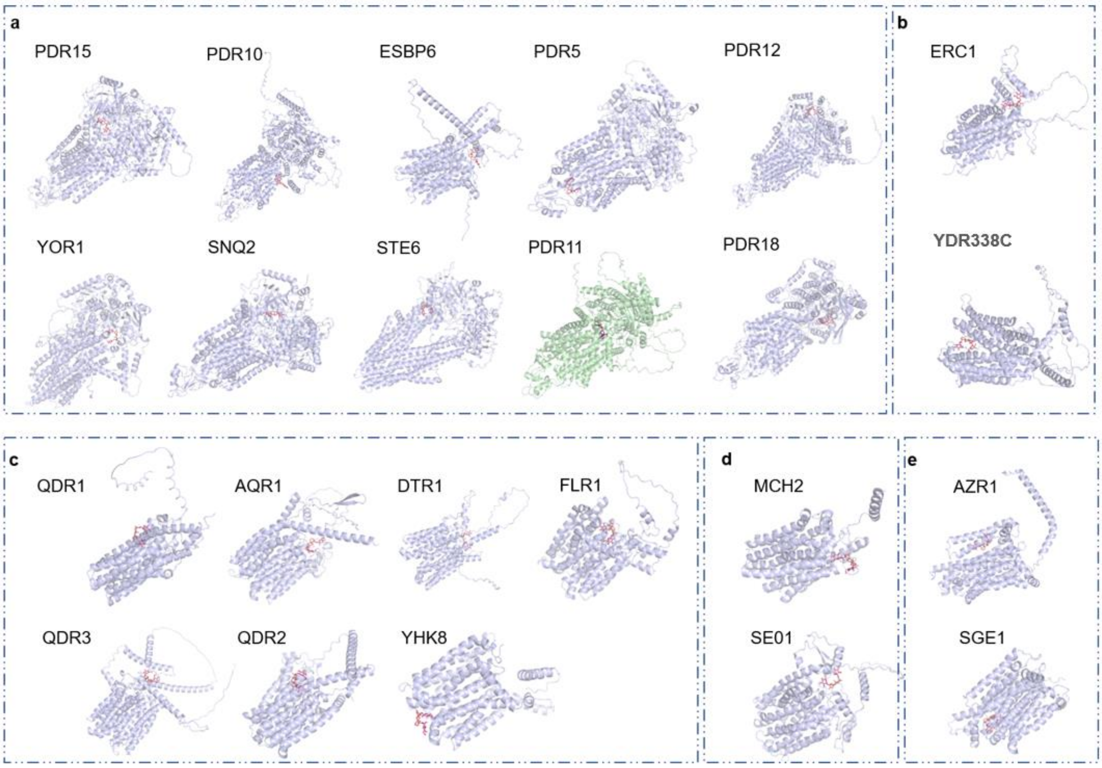

Supplementary Figure 11. The molecular docking results of membrane exporters with rubusoside by AutoDock software. The protein structures were from AlphaFold Protein Structure Database (https://alphafold.ebi.ac.uk/). Image analysis was carried out on the Pymol software. **a.** Overall structure of ATP-binding cassette (ABC) docking with rubusoside. **b.** Overall structure of Multidrug and toxic compound extrusion (MATE) docking with rubusoside. **c.** Overall structure of Major Facilitator Superfamily (MFS) docking with rubusoside. **d.** Overall structure of Probable exporters docking with rubusoside. **e.** Overall structure of resistance exporters docking with rubusoside.

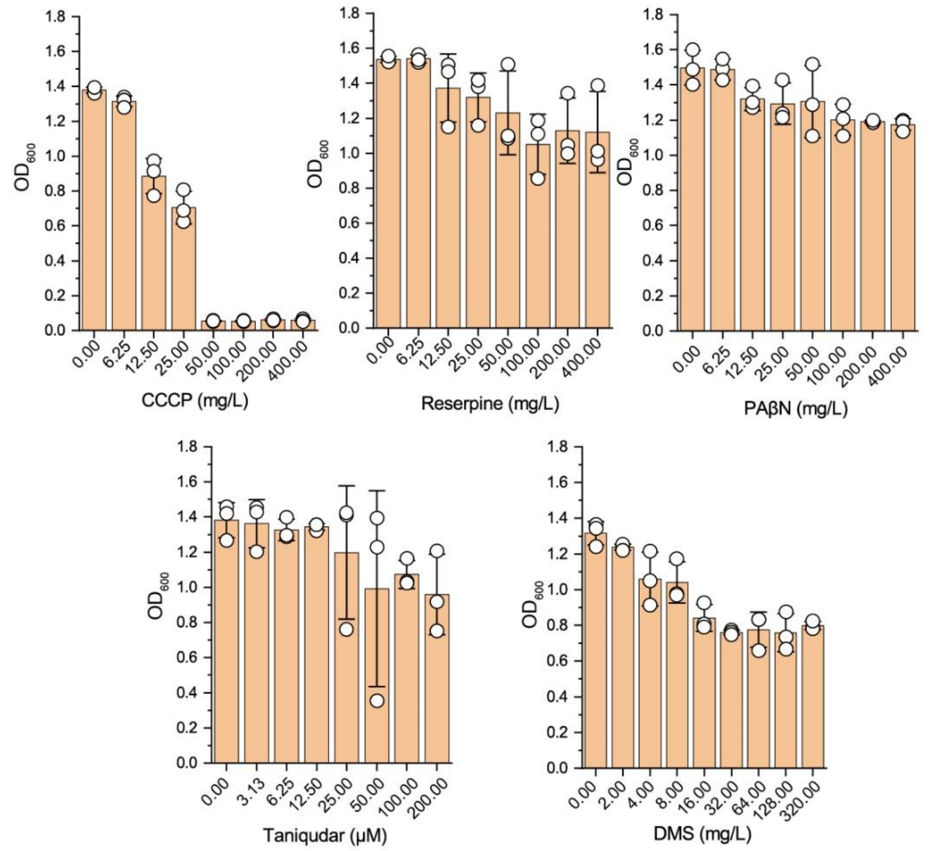

Supplementary Figure 12. The MIC of ABC transporter inhibitors to yeast. Carbonyl CCCP: Cyanide m-Chlorophenylhydrazone; PAβN: phenyl-arginine-β-naphthylamide; DMS: dexamethasone. All the strains were cultured in 96-well plates, and grown at 30°C with shaking at 750 rpm. The cell optical density (OD, excitation, 600 nm) was detected by a microplate reader (BioTek). Three experiments (n = 3) were repeated independently with similar results. Data are presented as mean values ± SD.

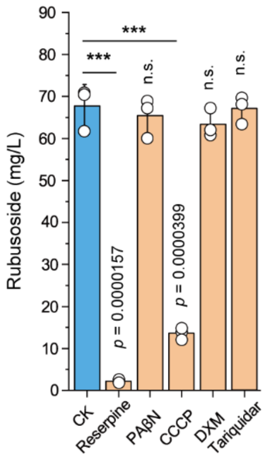

Supplementary Figure 13. Weakening the rubusoside exportation by ABC transporter inhibitors. Carbonyl CCCP: Cyanide m-Chlorophenylhydrazone; PAβN: phenyl-arginine-β-naphthylamide; DMS: dexamethasone. Three experiments (n = 3) were repeated independently with similar results. Data are presented as mean values ± SD. The statistical evaluation (*p*-value) was performed by two-sided t-test, *$p < 0.05$, **$p < 0.01$, ***$p < 0.001$, and n.s. presents $p > 0.05$.

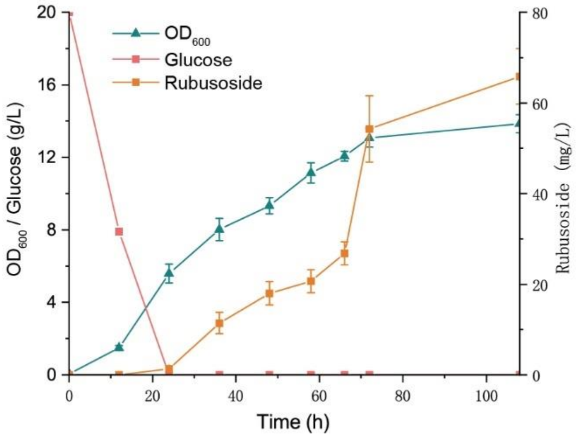

Supplementary Figure 14. The relationship of rubusoside production with the glucose concentration and fermentation time in SGN08 strain. Three experiments (n = 3) were repeated independently with similar results. Data are presented as mean values ± SD.

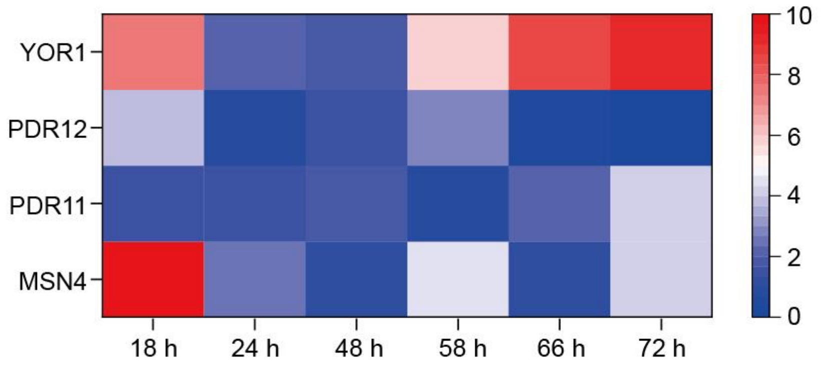

Supplementary Figure 15. Reverse-transcription-polymerase chain reaction (RT-qPCR) results of the ABC transporters (YOR1, PDR12, and PDR11) and the stress-responsive regulator MSN4 with the rubusoside accumulation at different fermentation times in SGN08 strain.

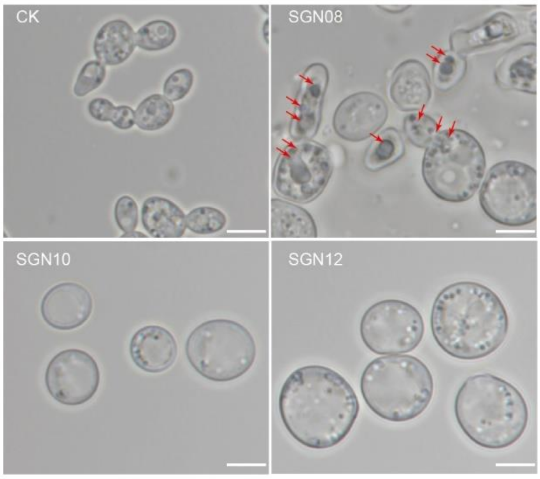

Supplementary Figure 16. Micrographs of the original *S. cerevisiae* and the rubusoside producing cells. Strains were cultivated in shake-flask, and cells were collected after fermentation 48 h by microscopy immersion oil (100×). Bar = 5 μm. Three experiments (n = 3) were repeated independently with similar results. Image analysis was carried out on the ImageJ 1.53k software. CK: the original *Saccharomyces cerevisiae* CEN.PK2-1C.

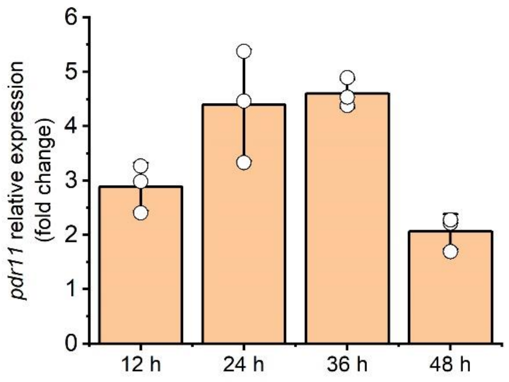

Supplementary Figure 17. Expression fold changes of *PDR11* in SGN12 strain compared with SGN08 strain. RT-qPCR was applied to detected whether the efflux pump *pdr11* can be up-regulated after MSN4 overexpressed. Three experiments (n = 3) were repeated independently with similar results. Data are presented as mean values ± SD.

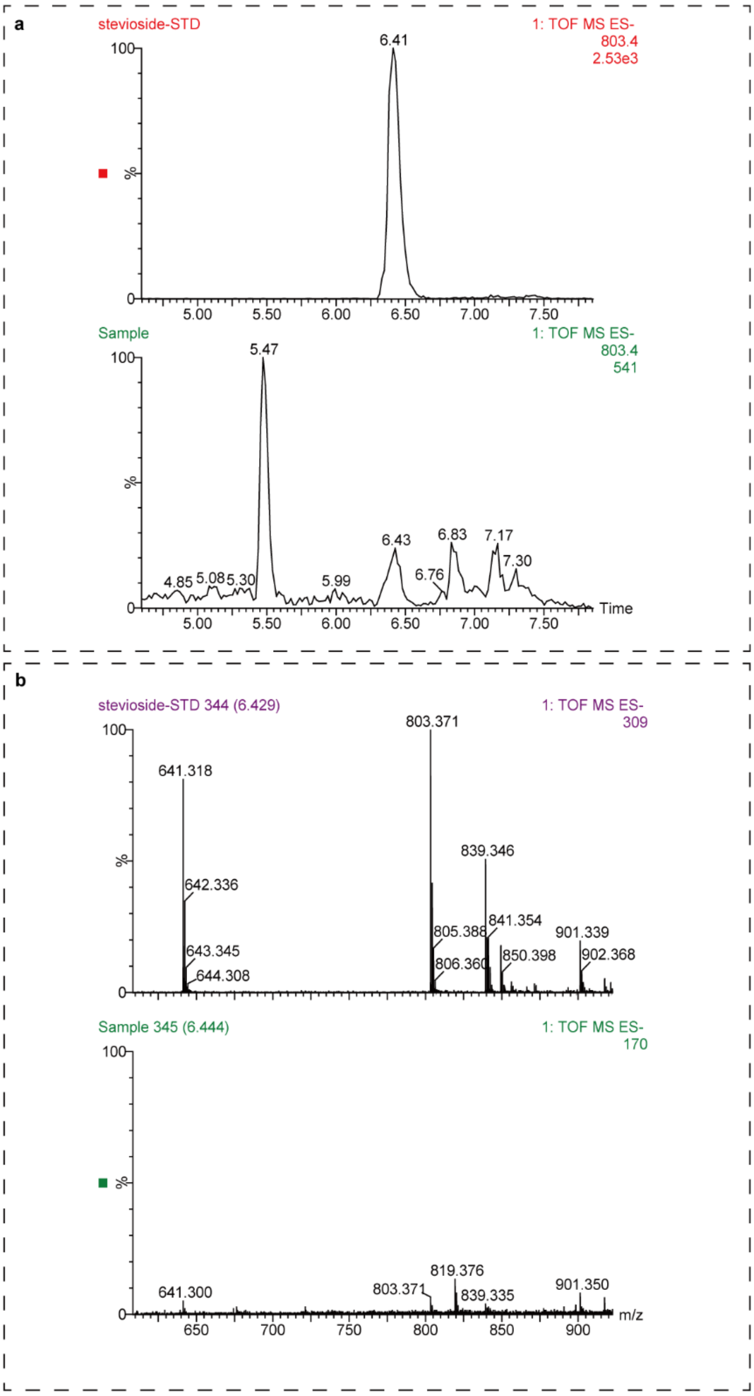

Supplementary Figure 18. The LC-MS/MS results of M04 strain. a. HPLC spectra of stevioside and the standard. b. MS/MS analysis results of Reb A and the standard in negative ion mode. The m/z of stevioside is 803.4. $[M + Cl]^-$ = 839.3. $[M - H]^-$ = 803.4. $[M – 1Clucose]^-$ = 641.3.

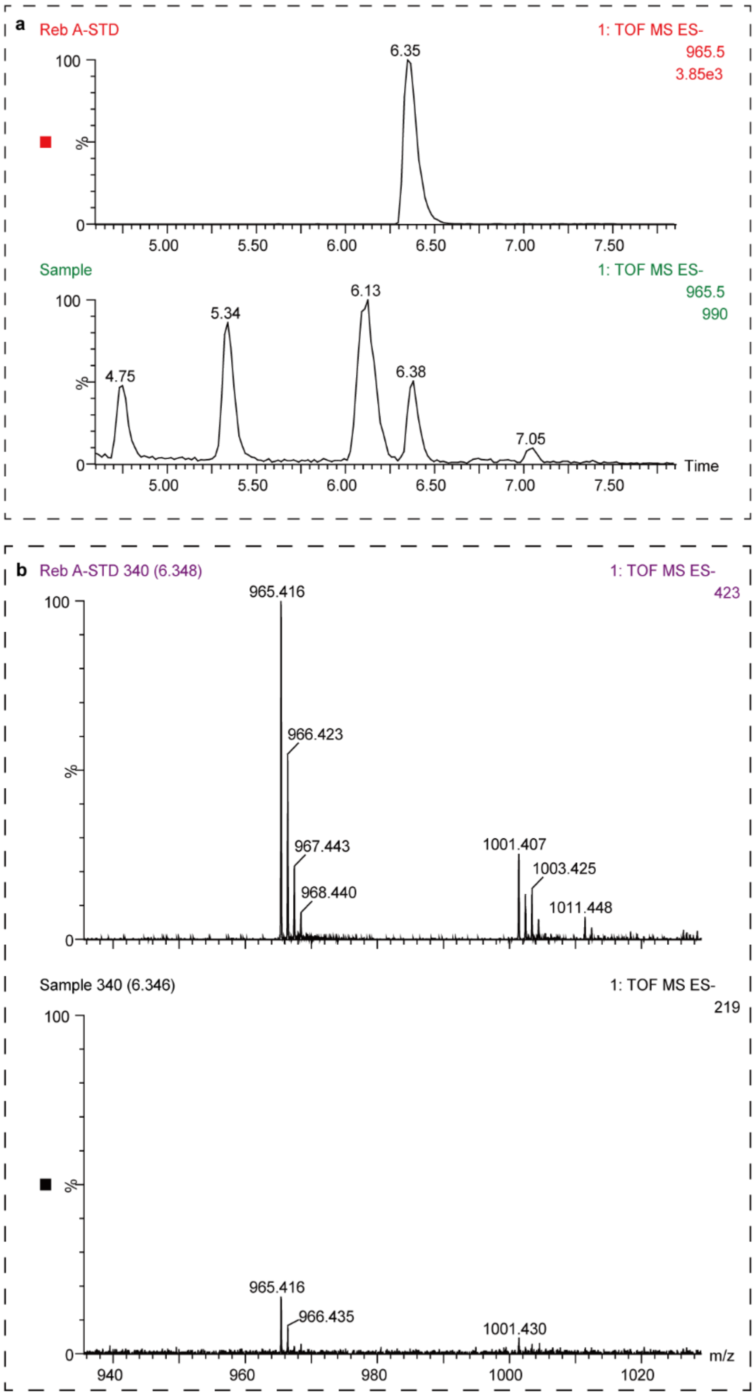

Supplementary Figure 19. The LC-MS/MS results of M04 strain. a. HPLC spectra of Reb A and the standard. b. MS/MS analysis results of Reb A and the standard in negative ion mode. The m/z of Reb A is 965.4. $[M + Cl]^-$ = 1001.4. $[M - H]^-$ = 965.4

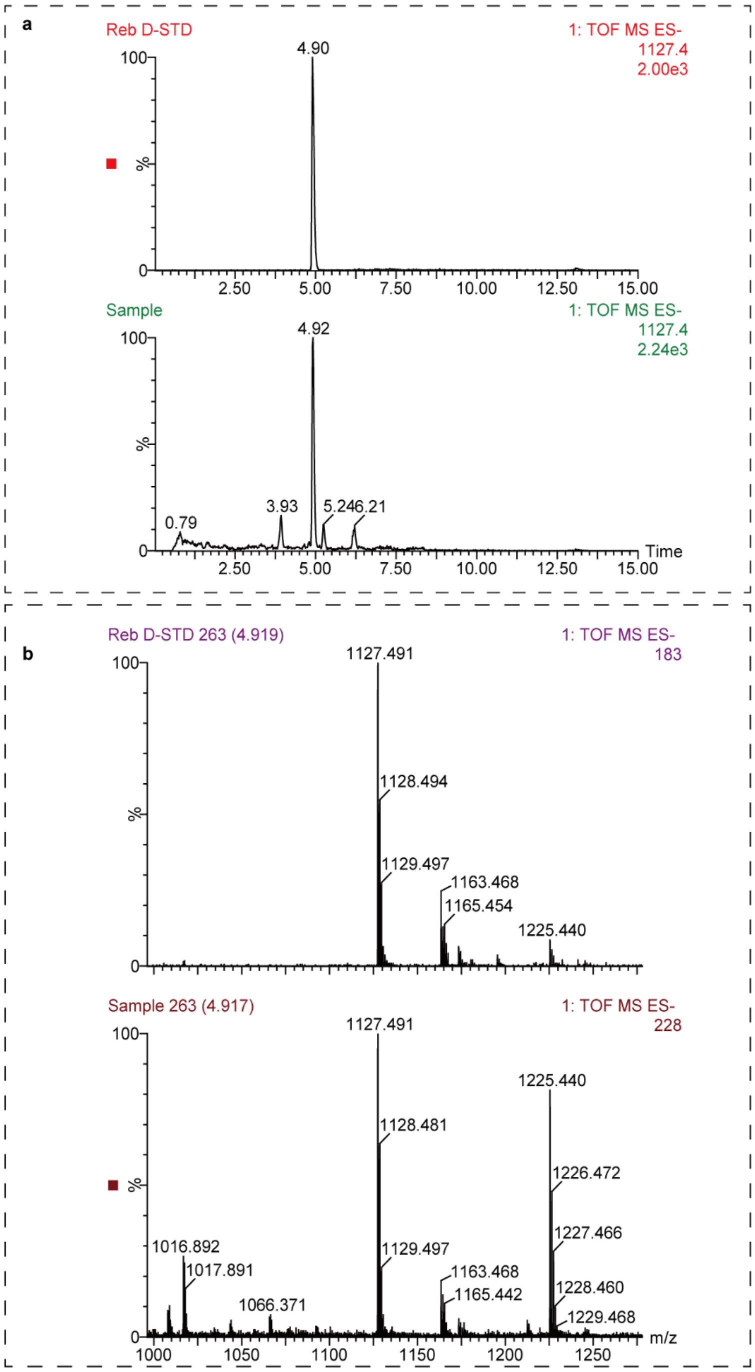

Supplementary Figure 20. The LC-MS/MS results of M04 strain. a. HPLC spectra of Reb D and the standard. b. MS/MS analysis results of Reb D and the standard in negative ion mode. The m/z of Reb D is 1127.5. $[M + Cl]^-$ = 1163.5. $[M - H]^-$ = 1127.5.

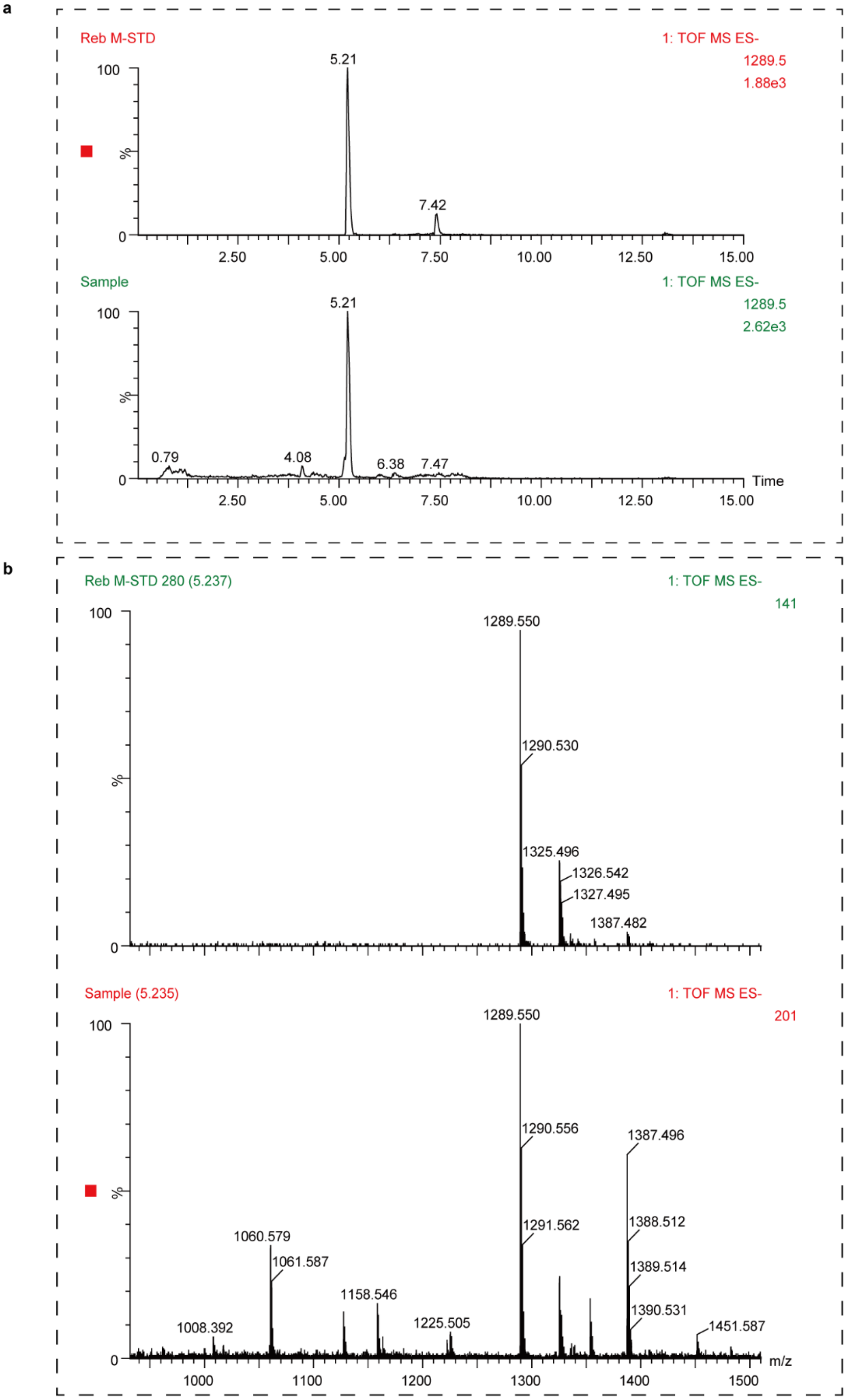

Supplementary Figure 21. The LC-MS/MS results of M04 strain. a. HPLC spectra of Reb M and the standard. b. MS/MS analysis results of Reb M and the standard in negative ion mode. The m/z of Reb M is 1289.6. [M + Cl]$^-$ = 1325.5, [M - H]$^-$ = 1289.6.

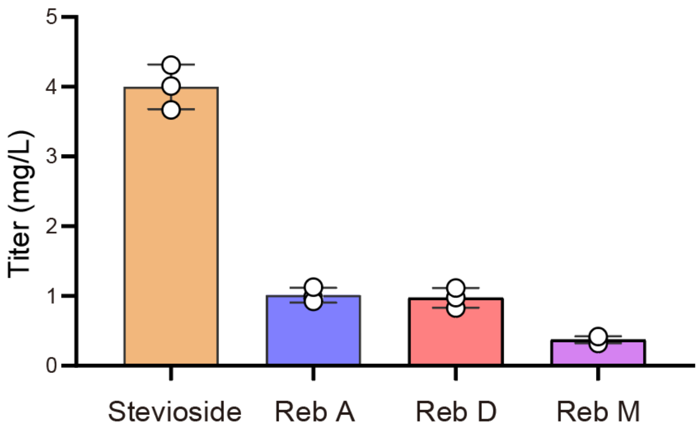

Supplementary Figure 22. The titers of stevioside, Reb A, Reb D, and Reb M in M04 strain.

The rebaudiosides titer was lower than 1 mg/L. Three experiments (n = 3) were repeated independently with similar results. Data are presented as mean values ± SD.

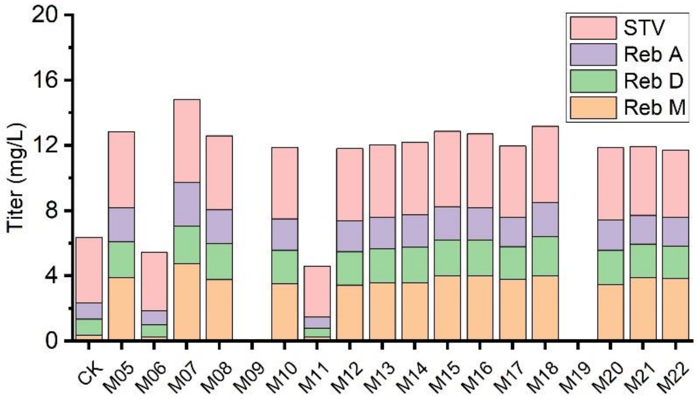

Supplementary Figure 23. The titers change of STV, Reb A, Reb D, and Reb M via engineering EUGT11 based on Rosetta Cartesian_ddg results. CK stands for M04 strain.

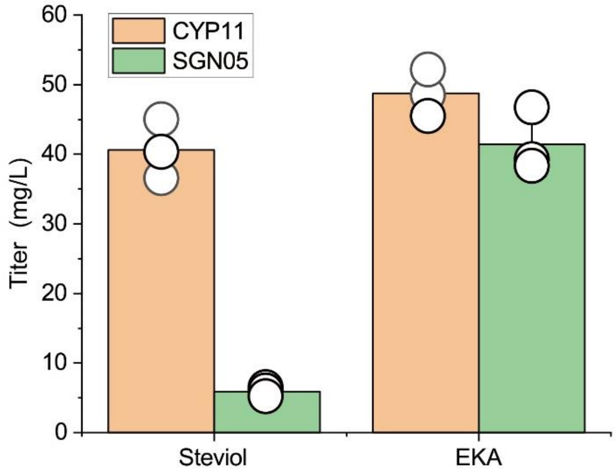

Supplementary Figure 24. The steviol and EKA titer changes in the strain CYP11 and SGN05 via engineering P450s. Three experiments (n = 3) were repeated independently with similar results. Data are presented as mean values ± SD.

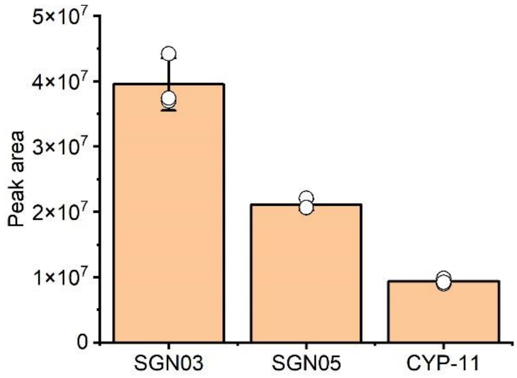

Supplementary Figure 25. Accumulation changes of *ent*-kaurene after intruding and optimizing the P450s Module. Three experiments (n = 3) were repeated independently with similar results. Data are presented as mean values ± SD.

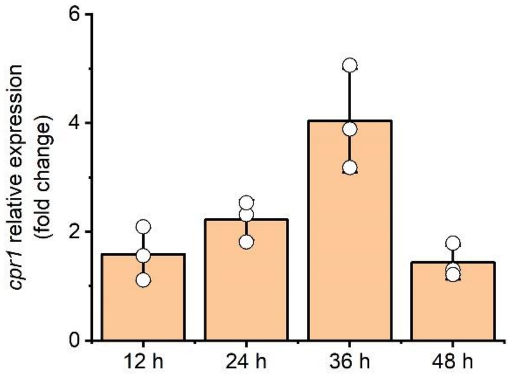

Supplementary Figure 26. RT-qPCR to detected the relative expression fold changes of trCPR1 to CPR1. Three experiments (n = 3) were repeated independently with similar results. Data are presented as mean values ± SD.

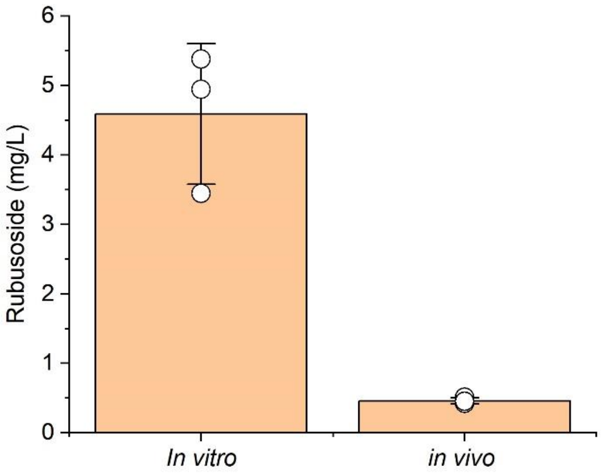

Supplementary Figure 27. The rubusoside titer *in vivo* and *in vitro* when PDR11 was deleted (SGN08-Δpdr11 strain). Three experiments (n = 3) were repeated independently with similar results. Data are presented as mean values ± SD.

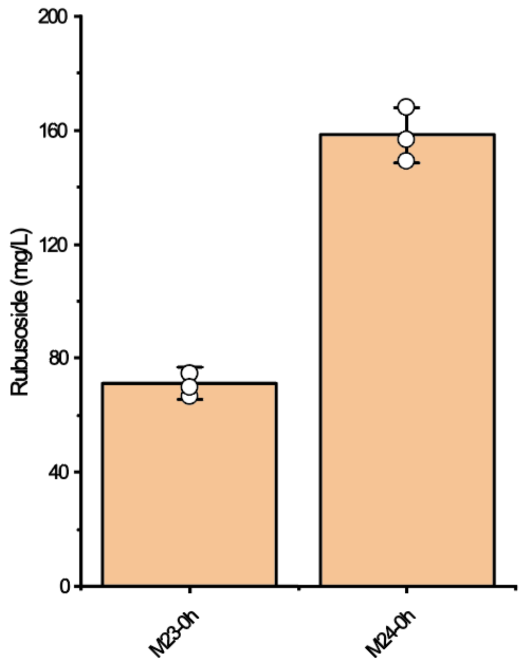

Supplementary Figure 28. The rubusoside titer changes in M24 strain via overexpression MSN4 in M23 strain. Three experiments (n = 3) were repeated independently with similar results. Data are presented as mean values ± SD.

Supplementary Methods:

Determination of minimum inhibitory concentration (MIC).

Minimum inhibitory concentrations (MICs) of the ABC transporters inhibitors were determined referring to the broth microdilution method[1]. Five common inhibitors were selected to destroy the ABC transporters function in yeast, including reserpine, Carbonyl Cyanide m-Chlorophenylhydrazone (CCCP), PAβN, tariquidar, and dexamethasone (DMS). Strains were tested in YPD using 96-well plates, with an inoculum of approximately 10^4 CFU/mL. The yeast growth was recorded after 18 to 24 h of incubation at 30°C by measuring OD at 600 nm using Cytation microplate reader (BioTek). The lowest concentration totally preventing growth was identified as the MIC. According to the MIC, the inhibitors were added to medium to detect the extracellular rubusoside titer in the SGN08 strain. Each of the tests was done in triplicate.

Reverse Transcription-Polymerase Chain Reaction.

RT-qPCR was used to analyze the change of genes transcriptional level. Based on the rubusoside synthesis time (Fig. S31), the strain was collected at 18 h, 24 h, 48 h, 58 h, 66 h, and 72 h. The yeast cell wall was broken by snailase (Sangon Biotech) RNA was extracted using RNA simple Kit (TIANGEN Biotech) following the manufacturer's instructions. Reverse transcription was conducted using a PrimeScript™ RT reagent Kit with gDNA Eraser (TaKaRa). Quantitative PCR was performed on LightCycler480 system (Roche Applied Science) with SYBR®Premix Ex Taq™ II (Tli RNaseH Plus) (TaKaRa). The relative changes in mRNA level expression were calculated using the $2^{-\Delta\Delta CT}$method, the 18S rRNA was selected as the reference gene. Each of the tests was done in triplicate.

Computational analysis.

The structure of EUGT11 was modelled using Alphafold2[2], and refined by Rosetta Relax[3–6]. Proline scan was conducted using Rosetta Cartesian_ddg[7] based on the energy minimized structure of EUGT11. The results may be caused by the low catalytic activity of the wild-type EUGT11, and which cannot high-efficiently transform the rubusoside into stevioside. To improve the catalytic efficiency, the EUGT11 was engineered. The Proline scan was conducted by Rosetta Cartesian_ddG, and the EUGT11 structure was modeled by I-tasser and refined by Rosetta Relax precede to Cartesian_ddG prediction. Eighteen residues (R255, D408, C121, A72, Y9, D258, S282, A272, T169, S152, A323, A349, A153, G198, A399, H132, S49, and E434) displayed free energy change below 1 kcal/mol, which suggested the increased thermostability.

Statistical analysis.

All experiments were independently carried out at least three times, and the results were expressed as mean ± standard deviation (SD). All the statistical evaluation (*p*-value) was performed by two-sided t-test, $^{*}p< 0.05$, $^{**}p< 0.01$, $^{***}p< 0.001$, and $p> 0.05$ is presented by no significance (n.s.).

Supplementary References:

1. Jiang, X. *et al.* Efflux pump-mediated benzalkonium chloride resistance in Listeria monocytogenes isolated from retail food. *Int. J. Food. Microbiol.* **217**, 141–145 (2016).
2. Senior, A. W. *et al.* Improved protein structure prediction using potentials from deep learning. *Nature*. **577**, 706–710 (2020).
3. Nivón, L. G., Moretti, R. & Baker, D. A Pareto-optimal refinement method for protein design scaffolds. *PloS. One*. **8**, e59004 (2013).
4. Conway, P., Tyka, M. D., DiMaio, F., Konerding, D. E. & Baker, D. Relaxation of backbone bond geometry improves protein energy landscape modeling. *Protein. Sci. Publ. Protein. Soc.* **23**, 47–55 (2014).
5. Khatib, F. *et al.* Algorithm discovery by protein folding game players. *Proc. Natl. Acad. Sci. U. S. A.* **108**, 18949–18953 (2011).
6. Tyka, M. D. *et al.* Alternate states of proteins revealed by detailed energy landscape mapping. *J. Mol. Biol.* **405**, 607–618 (2011).
7. Park, H. *et al.* Simultaneous optimization of biomolecular energy functions on features from small molecules and macromolecules. *J. Chem. Theory. Comput.* **12**, 6201–6212 (2016).
