# Supplementary material for: De novo biosynthesis of rubusoside and rebaudiosides in engineered yeasts
Source: Nat Commun. 2022 Jun 1;13:3040. doi: 10.1038/s41467-022-30826-2 (PMC9160076; doi:10.1038/s41467-022-30826-2)
Supplement: Supplementary file 2 — Description of Additional Supplementary Files [file 41467_2022_30826_MOESM2_ESM.pdf]

Description of Additional Supplementary Files

File name: Supplementary Data 1

Description: the metabolomics methodology results of the M23 strain by Progenesis QI v2.4 software.

File name: Supplementary Data 2

Description:

Table 1: Heterologous gene sequences used in this study.

Table 2: Plasmids used in this work.

Table 3: Primers used in this work.

Table 4: Strains used in this work.
