# Supplementary material for: De novo biosynthesis of rubusoside and rebaudiosides in engineered yeasts
Source: Nat Commun. 2022 Jun 1;13:3040. doi: 10.1038/s41467-022-30826-2 (PMC9160076; doi:10.1038/s41467-022-30826-2)
Supplement: Supplementary file 5 — Reporting Summary [file 41467_2022_30826_MOESM5_ESM.pdf]

nature portfolio

Corresponding author(s): Long Liu

Last updated by author(s): May 4, 2022

Reporting Summary

Nature Portfolio wishes to improve the reproducibility of the work that we publish. This form provides structure for consistency and transparency in reporting. For further information on Nature Portfolio policies, see our Editorial Policies and the Editorial Policy Checklist.

Statistics

For all statistical analyses, confirm that the following items are present in the figure legend, table legend, main text, or Methods section.

n/a	Confirmed	
☐	☒	The exact sample size (n) for each experimental group/condition, given as a discrete number and unit of measurement
☐	☒	A statement on whether measurements were taken from distinct samples or whether the same sample was measured repeatedly
☐	☒	The statistical test(s) used AND whether they are one- or two-sided<br>*Only common tests should be described solely by name; describe more complex techniques in the Methods section.*
☒	☐	A description of all covariates tested
☒	☐	A description of any assumptions or corrections, such as tests of normality and adjustment for multiple comparisons
☐	☒	A full description of the statistical parameters including central tendency (e.g. means) or other basic estimates (e.g. regression coefficient) AND variation (e.g. standard deviation) or associated estimates of uncertainty (e.g. confidence intervals)
☐	☒	For null hypothesis testing, the test statistic (e.g. F, t, r) with confidence intervals, effect sizes, degrees of freedom and P value noted<br>*Give P values as exact values whenever suitable.*
☒	☐	For Bayesian analysis, information on the choice of priors and Markov chain Monte Carlo settings
☒	☐	For hierarchical and complex designs, identification of the appropriate level for tests and full reporting of outcomes
☒	☐	Estimates of effect sizes (e.g. Cohen's d, Pearson's r), indicating how they were calculated

Our web collection on statistics for biologists contains articles on many of the points above.

Software and code

Policy information about availability of computer code

Data collection	Waters (MALDI SYNAPT MS) was used to collect the LC-MS/MS data, SHIMADZU (GCMS-QP2010Ultra) was used to collect the GC-MS data, Origin 2021 was used to collect the single particle data, Field emission scanning electron microscopy was collected by cold field emission scanning electron microscope observation (SU8220, HITACHI), fluorescence proteins were observed by Leica microscope (Leica, Mannheim, Germany) , The model used in this study with some modification is based on the model yeast 8.4.0 (https://github.com/SysBioChalmers/yeast-GEM/releases).
Data analysis	Excel (Microsoft Office 365) for quantitative analysis data in this paper, Leica LAS X software package and the Image J 1.53k software (National Institutes of Health, Bethesda, MA) were used to analyze visual image processing, and Matlab 2019b was used to analyze metabolite models, Gromacs-2020 was used to perform molecular dynamics simulation. the Progenesis QI v2.4 software (Waters) was used to obtain reliable and definitive Identity Documents of the SGs types.

For manuscripts utilizing custom algorithms or software that are central to the research but not yet described in published literature, software must be made available to editors and reviewers. We strongly encourage code deposition in a community repository (e.g. GitHub). See the Nature Portfolio guidelines for submitting code & software for further information.

Data

Policy information about availability of data

All manuscripts must include a data availability statement. This statement should provide the following information, where applicable:

- Accession codes, unique identifiers, or web links for publicly available datasets
- A description of any restrictions on data availability
- For clinical datasets or third party data, please ensure that the statement adheres to our policy

The authors declare that all data supporting the findings of this study are available in the article and its supplementary files or are available from the corresponding author on request. Sequence data in this article can be found in the National Coalition Building Institute (NCBI) under the accession codes and links of DNA sequences presented in the Source Data file. All the protein structures of efflux pumps in this study have been predicted previously and are obtained from Alpha fold; all the links of efflux pump protein structure are listed in Supplementary Figure 11 in Source Data file. The source data for all figures reported in the article and its supplementary information is provided in the Source Data file. The metabolomics results of the M23 strain are provided in Supplementary Data 1. Heterologous gene sequences, plasmids, primers and strains used in this work are listed in the Supplementary Table 1-4, provided in the Supplementary Data 2 file.

Field-specific reporting

Please select the one below that is the best fit for your research. If you are not sure, read the appropriate sections before making your selection.

[x] Life sciences [ ] Behavioural & social sciences [ ] Ecological, evolutionary & environmental sciences

For a reference copy of the document with all sections, see nature.com/documents/nr-reporting-summary-flat.pdf

Life sciences study design

All studies must disclose on these points even when the disclosure is negative.

Sample size	No sample-size calculations were performed. As indicated in the text, all experiments were performed from a single colony, which provides some limited information about the distribution of the measurements and is typical of similar experiments and studies in the field.
Data exclusions	There is no data exclusion in our study.
Replication	All the biochemical and biological experiments were performed in three replicated or more. Data were repeatable on different date.
Randomization	The samples of yeast cultures that were split into different conditions were random samplings, and there is no control over which cells will be selected. And pipet tips were used to scoop few cells for culturing, the scooping locations are all random. Randomization was used in some statistical test as described in the paper.
Blinding	There is no blinding in our study. We rationally designed and carried out the experiments to achieve our goal, and the data did not require blinding.

Reporting for specific materials, systems and methods

We require information from authors about some types of materials, experimental systems and methods used in many studies. Here, indicate whether each material, system or method listed is relevant to your study. If you are not sure if a list item applies to your research, read the appropriate section before selecting a response.

Materials & experimental systems

n/a	Involved in the study	
[x]	[ ]	Antibodies
[ ]	[x]	Eukaryotic cell lines
[x]	[ ]	Palaeontology and archaeology
[x]	[ ]	Animals and other organisms
[x]	[ ]	Human research participants
[x]	[ ]	Clinical data
[x]	[ ]	Dual use research of concern

Methods

n/a	Involved in the study	
[x]	[ ]	ChIP-seq
[x]	[ ]	Flow cytometry
[x]	[ ]	MRI-based neuroimaging

Eukaryotic cell lines

Policy information about cell lines

Cell line source(s)	S. cerevisiae CEN.PK2-1C was collected from multiple sources over the years, and stored in our lab.
Authentication	Genome sequencing
Mycoplasma contamination	No contamination.
Commonly misidentified lines (See ICLAC register)	None.
